# Supplementary material for: Learning inherent genetic patterns and trait associations with deep generative models for discrete genotype simulation
Source: Gigascience. 2026 Apr 14;15:giag044. doi: 10.1093/gigascience/giag044 (PMC13163187; doi:10.1093/gigascience/giag044)

GigaScience

# Learning Inherent Genetic Patterns and Trait Associations with Deep Generative Models for Discrete Genotype Simulation

--Manuscript Draft--

| | |
|---|---|
| **Manuscript Number:** | GIGA-D-25-00343 |
| **Full Title:** | Learning Inherent Genetic Patterns and Trait Associations with Deep Generative Models for Discrete Genotype Simulation |
| **Article Type:** | Research |
| **Funding Information:** | INRAE DigitBio Metaprogram / PhD student Sihan XIE |
| **Abstract:** | Background: Deep generative models open new avenues for simulating realistic genomic data while preserving privacy and addressing data accessibility constraints. While previous studies have primarily focused on generating gene expression or haplotype data, this study explores generating genotype data in both unconditioned and phenotype-conditioned settings, which is inherently more challenging due to the discrete nature of genotype data.<br>Results: We developed and evaluated commonly used generative models, including Variational Autoencoders (VAEs), Diffusion Models, and Generative Adversarial Networks (GANs), and proposed adaptation tailored to discrete genotype data. We conducted extensive experiments on large-scale datasets, including all chromosomes from cow and multiple chromosomes from human. Model performance was assessed using a well-established set of metrics drawn from both deep learning and quantitative genetics literature. Our results show that these models can effectively capture genetic patterns and preserve genotype–phenotype association.<br>Conclusions: As deep generative models are able to reproduce key characteristics of genotype data, they can serve as direct tools for genotype–phenotype simulation, while also enabling privacy-preserving data sharing. Our findings provide a comprehensive evaluation of these models and offer practical guidance for future research in genotype simulation. |
| **Corresponding Author:** | Sihan XIE<br>INRAE Centre Île-de-France Jouy-en-Josas Antony: Institut National de Recherche pour l'Agriculture l'Alimentation et l'Environnement Centre Ile-de-France Jouy-en-Josas Antony<br>Jouy-en-josas, FRANCE |
| **Corresponding Author Secondary Information:** | |
| **Corresponding Author's Institution:** | INRAE Centre Île-de-France Jouy-en-Josas Antony: Institut National de Recherche pour l'Agriculture l'Alimentation et l'Environnement Centre Ile-de-France Jouy-en-Josas Antony |
| **Corresponding Author's Secondary Institution:** | |
| **First Author:** | Sihan XIE |
| **First Author Secondary Information:** | |
| **Order of Authors:** | Sihan XIE<br>Thierry Tribout<br>Didier Boichard<br>Blaise Hanczar<br>Julien Chiquet<br>Eric Barrey |
| **Order of Authors Secondary Information:** | |
| **Additional Information:** | |

Powered by Editorial Manager® and ProduXion Manager® from Aries Systems Corporation

| Question | Response |
| --- | --- |
| Are you submitting this manuscript to a special series or article collection? | No |
| **Experimental design and statistics**<br><br>Full details of the experimental design and statistical methods used should be given in the Methods section, as detailed in our Minimum Standards Reporting Checklist. Information essential to interpreting the data presented should be made available in the figure legends.<br><br>Have you included all the information requested in your manuscript? | Yes |
| **Resources**<br><br>A description of all resources used, including antibodies, cell lines, animals and software tools, with enough information to allow them to be uniquely identified, should be included in the Methods section. Authors are strongly encouraged to cite Research Resource Identifiers (RRIDs) for antibodies, model organisms and tools, where possible.<br><br>Have you included the information requested as detailed in our Minimum Standards Reporting Checklist? | Yes |
| **Availability of data and materials**<br><br>All datasets and code on which the conclusions of the paper rely must be either included in your submission or deposited in publicly available repositories (where available and ethically appropriate), referencing such data using a unique identifier in the references and in the "Availability of Data and Materials" section of your manuscript. | Yes |

| | |
|---|---|
| Have you have met the above requirement as detailed in our Minimum Standards Reporting Checklist? | |
| GigaScience has policies and guidelines in place for the use of generative AI-writing tools such as ChatGPT. If you have used such writing tools to assist with writing the manuscript this must be declared and cited in the text. Authors should not list AI-writing tools and other AI-assisted technologies as an author or co-author and should acknowledge that they are fully responsible for text generated or refined by AI-writing tools.<p><br><br>A summary of use (particularly in the introduction or among methods) needs to be included at the end of the paper, and the outputs should also be included as a supplementary file hosted in GigaDB or other open repositories. Please <a href=https://academic.oup.com/gigascience/pages/editorial_policies_and_reporting_standards target="_new" > read our guidelines for more information. </a> <p><br><br>By submitting to GigaScience, you are aware of the journal's AI-writing tools policy, and if you have declared use of such tools below, you have acknowledged this where appropriate in your manuscript and have made a summary of use and outputs available. </b><p><br><b>AI-assisted writing tools have been used in the preparation of this manuscript? | No |

Latex main file

Click here to access/download;LaTeX - Main Document (TeX file);main.tex

Placeholder for OUP logo oup.pdf

Placeholder for journal logo gigascience-logo.pdf

*GigaScience*, 2025, 1–12

doi: xx.xxxx/xxxx
**Manuscript in Preparation**
**Paper**

PAPER

# Learning Inherent Genetic Patterns and Trait Associations with Deep Generative Models for Discrete Genotype Simulation

Sihan Xie[1,*], Thierry Tribout[1], Didier Boichard[1], Blaise Hanczar[2,†], Julien Chiquet[3,†] and Eric Barrey[1,†]

[1]Université Paris-Saclay, INRAE, AgroParisTech, GABI, 78350, Jouy-en-Josas, France and [2]Université Paris-Saclay, Univ Evry, IBISC, 91020, Evry-Courcouronnes, France and [3]Université Paris-Saclay, AgroParisTech, INRAE, UMR MIA Paris-Saclay, 91120, Palaiseau, France

[*] Corresponding author: sihan.xie@inrae.fr
[†] These authors contributed equally to this work.

## Abstract

**Background:** Deep generative models open new avenues for simulating realistic genomic data while preserving privacy and addressing data accessibility constraints. While previous studies have primarily focused on generating gene expression or haplotype data, this study explores generating genotype data in both unconditioned and phenotype-conditioned settings, which is inherently more challenging due to the discrete nature of genotype data.
**Results:** We developed and evaluated commonly used generative models, including Variational Autoencoders (VAEs), Diffusion Models, and Generative Adversarial Networks (GANs), and proposed adaptation tailored to discrete genotype data. We conducted extensive experiments on large-scale datasets, including all chromosomes from cow and multiple chromosomes from human. Model performance was assessed using a well-established set of metrics drawn from both deep learning and quantitative genetics literature. Our results show that these models can effectively capture genetic patterns and preserve genotype–phenotype association.
**Conclusions:** As deep generative models are able to reproduce key characteristics of genotype data, they can serve as direct tools for genotype–phenotype simulation, while also enabling privacy-preserving data sharing. Our findings provide a comprehensive evaluation of these models and offer practical guidance for future research in genotype simulation.

**Key words**: Deep Generative Models, Genotype Simulation, Quantitative Genetics

## Introduction

The development of dense genotyping platforms and high-throughput sequencing technologies has significantly advanced genetic analysis [1, 2]. Today, genomic studies rely on large biobanks that contain vast amounts of genomic data. However, working with such datasets presents several challenges, including high sequencing costs, substantial storage requirements, privacy concerns, and access restrictions that limit data sharing. To address these issues, simulation tools and synthetic data are commonly used. Traditional statistical simulation methods are based on evolutionary models like Wright-Fisher model [3] and coalescent theory [4–6], where users need to specify evolutionary parameters or provide ancestral population. While these simulation tools [7–12] are powerful, they often simplify various aspects of population genetics, which may not fully capture the complexities of real-world datasets.

Recently, data-driven simulation methods based on deep generative models have gained attention in genomics. These approaches eliminate the need to explicitly specify genetic parameters by learning directly from data, enabling the reproduction of fine-scale ge-

**Compiled on:** August 27, 2025.
Draft manuscript prepared by the author.

nomic characteristics presented in the given population. By shifting from explicit genomic sequences to generative models, the genome-wide data remains private, while the trained models can be shared publicly without directly exposing individual-level genetic information.

Previous studies have applied generative models to various genomic modalities: [13–15] focused on gene expression data, [16, 17] focused on DNA sequence, and there is a substantial body of literature on haplotype data [18–25]. In this work, we propose a new study on genotype data, which represents genetic variation at specific positions in the genome known as Single Nucleotide Polymorphisms (SNPs). Unlike binary-valued haplotype, genotype for diploid organisms includes three possible values (0, 1, 2), representing the number of alternative alleles inherited from both parents, which introduces specific modeling challenges. Importantly, directly simulating genotype data offers several advantages over haplotype-based approaches: Haplotype-based simulation methods lack rich conditioning capabilities, whereas our method allows conditioning on phenotypic traits, enabling more flexible and application-oriented use cases. Furthermore, traditional genotype–phenotype simulation workflows via haplotypes involve a multi-step pipeline and our approach consolidates this into a single generative step, reducing complexity and avoiding additional modeling assumptions. Finally, haplotype simulators are limited to genomic regions with strong linkage disequilibrium (LD) on a single chromosome, whereas our study supports genome-wide simulation, demonstrated in our experiments on cow dataset where we modeled genotypes across all 29 autosomes simultaneously, significantly extending the scale of genomic data simulation.

This paper investigates the use of deep generative models for simulating genotype data, potentially conditioned on phenotype. Specifically, we adapt models such as Variational Autoencoders (VAEs) [26], Generative Adversarial Networks (GANs) [27], and Diffusion Models [28] to accommodate the discrete nature of genotype. Properly evaluating synthetic genotype is a critical aspect of our study, as the evaluation metrics commonly used in the Generative AI community, such as precision and recall [29], have never been applied in previous haplotype generation studies. We propose a comprehensive evaluation framework that integrates both deep learning and quantitative genetics approaches, providing a rigorous comparison of the reviewed models. The paper first introduces the generative models adapted for genotype data, followed by a description of our proposed evaluation framework. We then detail the experimental setup and present the main results. Finally, we discuss how the proposed models can be practically implemented, along with potential challenges and future research directions.

## Generative Models for Genotype

Building on recent advances in haplotype generation [19, 22–25], we adopt generative models such as Variational Autoencoders (VAEs) [26], Generative Adversarial Networks (GANs) [27], and diffusion models [28]. They are well-suited for capturing global dependencies across all SNPs, as opposed to relying on sequential autoregressive decomposition, which may not align with the underlying biological structure. Since genotype is a discrete sequence represented as $\mathbf{x} \in \{0, 1, 2\}^n$, we propose adaptation to better handle this structure.

### Variational Autoencoders

Variational Autoencoders (VAEs) [26] learn to approximate the underlying data distribution by introducing a latent variable. A VAE consists of two neural networks, parameterized by $\phi$ and $\theta$: an encoder that maps the input data $x$ to a latent representation $z$ via the approximate posterior $q_\phi(z \mid x)$, and a decoder that reconstructs $x$ from $z$ via the likelihood $p_\theta(x \mid z)$. The model is trained by maximizing the Evidence Lower BOund (ELBO) on the marginal likelihood $\log p(x)$, using the reparameterization trick to enable efficient gradient-based optimization. The ELBO is given by

$$\mathcal{L}(\theta, \phi; x) = \underbrace{\mathbb{E}_{q_\phi(z|x)}\left[\log p_\theta(x \mid z)\right]}_{\text{decoder for reconstruction}} - \underbrace{D_{\text{KL}}\left(q_\phi(z \mid x) \,\|\, p(z)\right)}_{\text{encoder for prior matching}}, \quad (1)$$

where $p(z)$ is the prior on the latent variable and $D_{\text{KL}}$ denotes the Kullback-Leibler divergence. Optimizing this objective encourages the model to learn a meaningful, structured latent space that can be sampled to generate new, realistic data. Specifically, new samples are obtained by drawing a latent vector $z$ from the prior and passing it through the decoder.

### Diffusion Models

Diffusion models (DMs), and in particular Denoising Diffusion Probabilistic Models (DDPMs) [28], can be viewed as a Markovian hierarchical VAE where each latent $x_t$ has the same dimension as the data $x_0$ and the encoder is not learned but is a fixed Gaussian noising process. During the encoding phase, also called the forward diffusion process, we gradually add Gaussian noise to input $x_0$ until it becomes pure noise $x_T$ over $T$ steps via a Markov chain:

$$q(x_t \,|\, x_{t-1}) = \mathcal{N}(\sqrt{\alpha_t}\, x_{t-1},\ \beta_t \mathbf{I}), \quad \alpha_t = 1 - \beta_t \quad \text{for } t = 1, \ldots, T. \quad (2)$$

Because of the Markov property, the Gaussian transition, and the independence of the noise at every step, one can collapse all $t$ steps into a single closed-form marginal:

$$q(x_t \,|\, x_0) = \mathcal{N}\Big(x_t;\ \sqrt{\bar{\alpha}_t}\, x_0,\ (1 - \bar{\alpha}_t)\mathbf{I}\Big), \quad \bar{\alpha}_t = \prod_{s=1}^{t} \alpha_s. \quad (3)$$

Intuitively, the hyperparameter $\beta_t$ controls the amount of noise injected at step $t$, and $\alpha_t = 1 - \beta_t$ is the fraction of signal retained. Our goal is to undo the added noise by learning $p_\theta(x_{t-1} \mid x_t)$, so that starting from $x_T \sim \mathcal{N}(0, I)$ we can step-by-step recover $x_0$. The true reverse $q(x_{t-1} \,|\, x_t)$ is intractable, but during training we know $x_0$. Hence, we can write down the exact one-step posterior as a Gaussian distribution with a closed-form mean $\mu_t(x_t, x_0)$ and variance $\sigma_t^2$:

$$q(x_{t-1} \mid x_t, x_0) = \mathcal{N}\Big(\frac{\sqrt{\alpha_t}\,(1 - \bar{\alpha}_{t-1})}{1 - \bar{\alpha}_t}\, x_t + \frac{\beta_t \sqrt{\bar{\alpha}_{t-1}}}{1 - \bar{\alpha}_t}\, x_0,\ \frac{\beta_t\,(1 - \bar{\alpha}_{t-1})}{1 - \bar{\alpha}_t}\,\mathbf{I}\Big). \quad (4)$$

For the reverse process, we learn to approximate the posterior in Equation 4. For the variance part, many implementations simply set $\sigma_t^2 = \beta_t$, which has a negligible loss on quality. For the mean part, since $\mu_t(x_t, x_0)$ requires the true $x_0$ which is unavailable at inference, the direct training objective is to predict $x_0$ given $x_t$ and $t$. In practice, however, it is more common and empirically more stable to train a network $\epsilon_\theta(x_t, t)$ to predict the injected noise at each timestep optimized via mean-squared error loss. Then using the predicted noise $\epsilon_\theta(x_t, t)$, we first infer an estimator of $x_0$, given by $\hat{x}_0 = \left(x_t - \sqrt{1 - \bar{\alpha}_t}\ \epsilon_\theta(x_t, t)\right) / \sqrt{\bar{\alpha}_t}$. Substituting $\hat{x}_0$ for $x_0$ in Equation 4 gives the familiar reverse-step update:

$$x_{t-1} = \frac{1}{\sqrt{\alpha_t}}\left(x_t - \frac{\beta_t}{\sqrt{1-\bar{\alpha}_t}}\, \epsilon_\theta(x_t, t)\right) + \sigma_t z, \quad z \sim \mathcal{N}(0, I). \quad (5)$$

Despite their success, DMs are not compatible with discrete data. Two main strategies have been proposed to address this limitation: (1) defining a diffusion-like process that operates in discrete space,

or (2) projecting the discrete input into a continuous latent space. Additionally, DMs can be computationally demanding during inference. To address both issues, we adopt the second strategy. Various methods can be used to construct a suitable latent space. For example, [16] employed a VAE to embed DNA sequences into a continuous representation. We follow the PCA-based approach originally developed for haplotype [24, 25]. Specifically, we project genotype into a lower-dimensional PCA space [30] and train the DMs in this continuous latent space. This single transformation yields three major benefits in one shot: it greatly reduces dimensionality and speeds up both training and inference; it transforms the discrete genotype into a continuous representation that matches the assumptions of DMs; And it allows precise reconstruction via a simple linear multiplication (Supplementary Section 1).

### Generative Adversarial Networks

While VAEs and DMs learn the data distribution by explicitly maximizing likelihood, Generative Adversarial Networks (GANs) [27] adopt a fundamentally different strategy. They avoid explicit density estimation by framing generation as a two-player game: a generator $G$ transforms a latent vector $z \sim p_Z$ (typically Gaussian) into a synthetic sample $G(z)$, and a discriminator $D$ attempts to distinguish real data from generated samples. Training proceeds by solving the minimax problem:

$$\min_G \max_D \underbrace{\mathbb{E}_{x\sim p_{\text{data}}}[\log D(x)] + \mathbb{E}_{z\sim p(z)}[\log(1 - D(G(z)))]}_{\text{binary cross-entropy loss}}. \quad (6)$$

Here, $D$ is trained as a binary classifier to assign high probability to real sample $x$ and low probability to generated sample $G(z)$, while $G$ is trained to fool $D$ by producing ever more realistic outputs.

From Equation 6, $G$ is trained by backpropagating gradients from $D$ through its outputs $G(z)$. This works when $G(z)$ is continuous but fails for discrete outputs, which break differentiability. In previous GAN-based haplotype generation studies [21–23], no specific treatment was proposed for this issue: $G$ output continuous values between 0 and 1, which were passed directly to $D$ during training. At inference, discrete values were recovered using a binarization threshold of 0.5. This binary setting can be interpreted in two ways. First, from a probabilistic perspective, $G$ outputs the probability of class 1, and inference chooses the most likely class. Motivated by this view, we tested a probabilistic approach: using a Softmax final layer in $G$ to predict class probabilities per SNP, and training $D$ to distinguish these from one-hot encoded real genotype sequences. However, this method produced unsatisfactory results. The second interpretation views binarization as a quantization operation that maps continuous outputs to a discrete set. We therefore explored several threshold-based strategies for our ternary genotype data, but observed suboptimal results (Supplementary Section 2). To enable end-to-end differentiable training on discrete outputs, we instead integrate a Gumbel-Softmax [31, 32] layer into $G$. The Gumbel-Softmax distribution provides a continuous approximation to categorical sampling by applying a temperature-controlled softmax to perturbed logits. Concretely, for each SNP, if the final layer of $G$ produces logits $\ell = (\ell_0, \ell_1, \ell_2)$, we then sample Gumbel noise $g_i \sim \text{Gumbel}(0, 1)$, and compute the relaxed one-hot vector

$$\tilde{p}_i = \frac{\exp((\ell_i + g_i)/\tau)}{\sum_{j=0}^{2} \exp((\ell_j + g_j)/\tau)}, \quad (7)$$

where $\tau$ is a temperature parameter. As $\tau \to 0$, $\tilde{p}_i$ becomes exactly one-hot vector. During training we anneal $\tau$ from a high initial value down toward 0 to balance exploration and discretization. At inference we take $\arg\max_i \tilde{p}_i$ to recover a discrete value in $\{0, 1, 2\}$.

### Wasserstein GANs with Gradient Penalty

GANs lack an explicit likelihood measure and can suffer from training instabilities such as mode collapse [33]. Subsequent refinements like Wasserstein GAN (WGAN) [34] was developed to address these issues. The original WGAN replaces the Jensen-Shannon divergence with the Earth-Mover (Wasserstein-1) distance by solving:

$$\min_G \max_{D\in\mathcal{D}} \mathbb{E}_{x\sim p_{\text{data}}}[D(x)] - \mathbb{E}_{z\sim p_Z}[D(G(z))], \quad (8)$$

where $\mathcal{D}$ is the set of 1-Lipschitz functions. While weight clipping was initially proposed to enforce the Lipschitz constraint, this approach is proved unstable in practice. The Wasserstein GAN with gradient penalty (WGAN-GP) [35] instead introduces an equivalent gradient penalty term that penalizes the deviation of the gradient norm from 1, leading to more stable training dynamics:

$$\min_G \max_D \underbrace{\mathbb{E}_{x\sim p_{\text{data}}}[D(x)] - \mathbb{E}_{z\sim p_Z}[D(G(z))]}_{\text{Wasserstein distance between real and synthetic}} - \lambda \underbrace{\mathbb{E}_{\hat{x}\sim p_{\hat{x}}}\left(\|\nabla_{\hat{x}} D(\hat{x})\|_2 - 1\right)^2}_{\text{Gradient penalty}}, \quad (9)$$

where $p_{\hat{x}}$ is the distribution of points interpolated between real and generated samples, and $\lambda$ controls the penalty strength.

### Conditional Generative Modeling

So far, we have focused on modeling the marginal distribution $p(x)$. In practice, it is more interesting to learn the conditional distribution $p(x \mid y)$, which provides control over the generated data through conditioning variable $y$. A straightforward approach is to append $y$ to $x$ as input during training [36]. At inference, we can sample from $p(x \mid y)$ by specifying a desired value of $y$ to guide generation.

In previous work on haplotype generation [19, 20, 25], models have frequently used ancestry group as conditioning variable to reflect population structure. For genotype, a natural choice is phenotype, particularly quantitative traits, which are often associated with genetic variation and enable the generation of synthetic population that mirror specific trait distribution.

## Evaluation Metrics for Synthetic Genotype

Genotype lacks the intuitive visual or semantic cues of images and text. We therefore propose a diverse set of metrics that provides a multi-faceted assessment.

### PCA and UMAP visualization

The PCA [30] and UMAP [37] projections provide an initial visual assessment of how well the synthetic population resembles the real one. These dimensionality reduction techniques highlight global structure and potential clustering patterns, offering a qualitative sense of alignment between the two distributions. However, they do not quantitatively measure distributional similarity and should be interpreted as complementary to more rigorous evaluation metrics.

### Genetic Parameters

#### *Allele Frequency and Genotype Frequency*

We compare allele and genotype frequencies [38] between real and synthetic cohorts as a basic sanity check. Let $N$ be the num-

ber of individuals, for a given SNP $i$, let $n_{2,i}$, $n_{1,i}$, and $n_{0,i}$ denote the counts of individuals with genotype 2, 1, and 0, respectively. The allele frequency at locus $i$ is $p_i = \left(2n_{2,i} + n_{1,i}\right)/(2N)$. The genotype frequency is the proportion of each genotype class, given by $f_i(2) = n_{2,i}/N$(homozygous alternative), $f_i(1) = n_{1,i}/N$(heterozygous), $f_i(0) = n_{0,i}/N$(homozygous reference). A strong concordance indicates that the model has accurately reproduced the per-locus marginal distribution, which is a prerequisite before assessing the higher order structure.

### *Aggregated Fixation Index*

The fixation index $F_{ST}$ [39, 40] is a widely used population genetic statistic that quantifies the degree of genetic differentiation among populations. It normalizes the difference between the total heterozygosity and the average heterozygosity within populations, yielding a value between 0 (no genetic differentiation) and 1 (complete genetic differentiation). For SNP $i$, let $p_{\text{real},i}$ and $p_{\text{syn},i}$ denote the allele frequencies in the real and synthetic cohorts respectively, assuming both cohorts are of the same size. Thus, the combined allele frequency for the total population is $p_{T,i} = \left(p_{real,i} + p_{syn,i}\right)/2$. Recall that for a given SNP $i$, the expected heterozygosity is given by $H = 1 - p^2 - (1-p)^2$. Thus, for the two subpopulations we have $H_{real,i} = 1 - p^2_{real,i} - (1 - p_{real,i})^2$ and $H_{syn,i} = 1 - p^2_{syn,i} - (1 - p_{syn,i})^2$. The average within-subpopulation heterozygosity is $H_{S,i} = (H_{real,i} + H_{syn,i})/2$. The heterozygosity in the combined population is $H_{T,i} = 1 - p^2_{T,i} - (1 - p_{T,i})^2$. The per-SNP fixation index is given by

$$F_{ST}(i) = \frac{H_{T,i} - H_{S,i}}{H_{T,i}}. \tag{10}$$

Recognizing that not all SNPs are equally informative, with those exhibiting higher heterozygosity providing greater insight into genetic diversity, we aggregate the per-SNP fixation index into a summary metric using a weighted average:

$$F_{ST}^{\text{aggregated}} = \frac{\sum_i H_{T,i} F_{ST}(i)}{\sum_i H_{T,i}} \tag{11}$$

### *Linkage Disequilibrium and Its Decay with Physical Distance Along Chromosome*

Linkage disequilibrium (LD) [41] measures the non-random association of alleles at different loci. Its decay with increasing physical distance along a chromosome reflects the effect of recombination in reshuffling genetic variation. For diploid genotype data, the unknown gametic phase complicates the accurate computation of LD statistics. To address this, we employ a fast estimator introduced in [42], which approximates LD between two loci without relying on the assumption of random mating or requiring iterative computation. This method is implemented in the *scikit-allel* Python library[1].

## Unsupervised Metrics for Structural Similarity

### *Precision and Recall*

Precision and recall, originally developed for classification, have been adapted to assess generative models [29]. Here, precision measures the quality of synthetic data by quantifying the fraction of generated samples that fall within the support of the real data distribution, while recall measures the diversity of synthetic data by quantifying the fraction of real samples that fall within the support of the synthetic data distribution. The F1 score is the harmonic mean of precision and recall.

To estimate the support of a data distribution, we define, for each sample in this dataset, a threshold $\epsilon$ as the distance to its $k^{th}$ nearest neighbor within the same set. This distance serves as the radius of a hypersphere centered on that sample, and the union of all such hyperspheres provides an estimate of the underlying manifold. Formally, let $R$ denote the set of real samples and $S$ the set of synthetic samples. Precision and recall are defined as follows:

$$\text{Precision} = \frac{1}{|S|} \sum_{s \in S} \mathbf{1}\left\{\exists\, r \in R \text{ such that } \|s - r\| < \epsilon_r\right\}, \tag{12}$$

$$\text{Recall} = \frac{1}{|R|} \sum_{r \in R} \mathbf{1}\left\{\exists\, s \in S \text{ such that } \|s - r\| < \epsilon_s\right\}. \tag{13}$$

In image-based applications, precision and recall are computed on high-level feature vectors extracted from pretrained VGG-16 classifier [43]. For genotype, no widely accepted pretrained network exists, we therefore use the original data directly for evaluation. For KNN-based manifold estimation, the $L_2$ distance is conventionally employed. Given that genotype is discrete, we experimented with both $L_1$ and $L_2$ distances and found no significant differences in the resulting metrics (Supplementary Section 3). We adopted $L_2$ distance for its greater computational efficiency. The choice of $k$ is crucial and we selected the value that yielded approximately 90% precision and recall on two real datasets.

### *Correlation Score*

To compare the moments of the real and synthetic distributions, correlation score is proposed in [13], the idea is to compute the Pearson correlation coefficient between the strictly upper-diagonal elements of the SNP-pairwise correlation matrices $M_{real}$ and $M_{syn}$:

$$\rho(M_{\text{real}}, M_{\text{syn}}) = \frac{2}{n(n-1)} \sum_{i=1}^{n-1} \sum_{j=i+1}^{n} \frac{M_{i,j;\text{real}} - \mu(M_{\text{real}})}{\sigma(M_{\text{real}})} \times \frac{M_{i,j;\text{syn}} - \mu(M_{\text{syn}})}{\sigma(M_{\text{syn}})}, \tag{14}$$

where $n$ is the number of SNPs, $\mu(M)$ is the mean and $\sigma(M)$ is the standard deviation of the strictly upper-diagonal elements.

## Supervised Metrics for Geno–Pheno Association

### *Genome-wide association study (GWAS)*

In quantitative genetics, Genome-Wide Association Study (GWAS) [44] is a fundamental tool for identifying genetic variants associated with specific traits. In GWAS, a per-SNP regression is performed and a two-sided t-test is used to determine whether the regression coefficient $\beta$ is significantly different from 0. The corresponding $p$-value gives us the significance of the association. GWAS can be viewed as a feature-importance method, since each SNP's estimated effect size $\beta$ and its $p$-value indicate how strongly that locus contributes to phenotype prediction. By comparing GWAS results obtained from synthetic population with those from real, we can directly evaluate whether our generative model has preserved key biological signals.

### *Phenotype Prediction Performance*

We further assess synthetic genotype by evaluating its utility in phenotype prediction. Specifically, we train an XGBoost and a multilayer perceptron (MLP) on synthetic data, then assess their performance on an independent real dataset not used during generative training. If a predictive model trained solely on synthetic data performs comparably to one trained on real data, it suggests that the synthetic population has faithfully preserved the underlying genotype–phenotype relationship.

1 https://scikit-allel.readthedocs.io/

## Privacy Leakage Assessment

***Nearest Neighbor Adversarial Accuracy (AA)***
Since genotype data is highly sensitive, our synthetic data must balance utility with privacy protection. We adopt the nearest neighbour adversarial accuracy (*AA*) proposed in [45], which is conceptually similar to precision and recall. Rather than estimating the entire manifold with a full KNN approach, we use 1NN to compare local neighborhood distances. The intuition is that synthetic data should be close enough to real data to preserve utility, yet not so close as to risk privacy leakage. For each real sample, we measure whether its distance to its nearest synthetic neighbour ($d_{RS}$) is larger than its distance to its nearest real neighbour ($d_{RR}$). Likewise, for each synthetic sample, we check whether its distance to its nearest real neighbour ($d_{SR}$) is larger than its distance to its nearest synthetic neighbour ($d_{SS}$). These comparisons yield two values: one for the real dataset ($AA_{real}$) and one for the synthetic dataset ($AA_{syn}$). The overall *AA* score is defined as the average of these two quantities. Formally, we have:

$$AA = \frac{1}{2}\Bigg( \underbrace{\frac{1}{N}\sum_{i=1}^{N} \mathbf{1}(d_{RS}(i) > d_{RR}(i))}_{AA_{\text{real}}} + \underbrace{\frac{1}{N}\sum_{i=1}^{N} \mathbf{1}(d_{SR}(i) > d_{SS}(i))}_{AA_{\text{syn}}} \Bigg). \tag{15}$$

Same as in the calculation of precision and recall, we use $L_2$ distance. An *AA* value near 0 indicates overfitting, while an *AA* value near 1 suggests underfitting. Ideally, an *AA* value around 0.5 reflects a good tradeoff between utility and privacy.

# Experimental Setting

The following section describes our experimental setup, including datasets, model architectures, hyperparameter choices, synthetic data simulation, and metric computation. A schematic overview is provided in Figure 1.

## Datasets

Since SNP frequency distributions and correlations vary across populations, techniques developed in one group may not generalize well to others. Therefore, we used two large-scale datasets from different species: a Holstein cow cohort and the human dataset from UK Biobank[2] [46]. For diploid organisms, genotypes were encoded as 0 (homozygous reference), 1 (heterozygous), and 2 (homozygous alternate).

**Cow:** Our cow dataset comprises 93,484 individuals genotyped at 50,161 SNPs across all 29 pairs of autosomes. The selected phenotype is fat content (FC), a milk production trait that reflects the proportion of fat in milk. Fat is a key component in dairy products and influences the taste, texture, and richness of milk. FC has relatively high heritability, estimated at approximately 0.50. For selection purpose, FC was analyzed with a mixed model that accounts for various fixed environmental effects, the permanent environmental effect of the cow, and the breeding value (Supplementary Section 4). The so-called Yield Deviations (YD) are therefore by-products of the French Holstein Single Step genomic evaluation [47, 48]. The YD of FC for a cow is the mean of its phenotypes that have been adjusted for all non-genetic effects estimated in the genetic evaluation, and serves as the conditioning phenotype in our study. We assessed model performance on two individual chromosomes: Chromosome 5 (2,238 SNPs), where the MGST1 gene [49] is located, and Chromosome 14 (1,771 SNPs), where the DGAT1 gene [50] is located. In our experiments, these chromosomes exhibited the strongest GWAS signals for the selected trait. We also evaluated the models using the full concatenated genotype across all chromosomes.

**Human:** UK Biobank provides genotype and phenotype data for 488,377 participants, including 805,426 variants comprising both SNPs and INDELs [51], across the 22 autosomes, sex chromosomes, and the mitochondrial chromosome. We used sex and height as conditioning phenotypes, as height is a highly heritable and polygenic trait [52]. Following the pipeline proposed in [53] to assemble our study subsets, we performed quality control using PLINK 1.9[3], including checks for sex discordance, individual and SNP missingness, minor-allele-frequency filtering, Hardy–Weinberg equilibrium testing, and LD-based tag SNP selection. Missing genotypes were imputed using Beagle 5.4[4]. To recover biologically relevant height loci, we incorporated annotations from Ensembl[5] and extracted 3,493 SNPs associated with height. In a final cohort of 291,023 individuals, we constructed 4 genotype datasets: the 3,493 height-associated variants; Chromosome 6 (12,283 SNPs) where a QTL was detected by GWAS; Chromosome 12 (9,780 SNPs) where IGF-1 gene [54] is located; a combined set of 42,409 SNPs from Chromosomes 3, 6, 12, and 17.

For VAE, GAN, and WGAN models, genotype was first transformed using one-hot encoding. For DM, we applied PCA and retained the number of principal components that captured 90% of the total variance in each dataset. Across all experiments, 70% of samples were used for training, 15% for validation, and 15% for testing.

## Models and Training

All models were implemented with fully connected layers. Because genotype sequences lack inherent spatial or temporal structure, we avoided convolutional and recurrent layers. Each model consisted of a sequence of dense layers, with layer widths heuristically scaled to the data dimension. To improve training stability and gradient flow, we incorporated residual connections [55]. The VAE employed a symmetric encoder–decoder architecture. The GAN and WGAN shared identical network architectures. In WGAN, the gradient penalty coefficient $\lambda$ was set to 10, with 5 discriminator updates per generator update. For DM, we tested several noise schedules and found that a linear $\beta$ schedule gave the best performance.

We performed a grid search over network architecture and key training hyperparameters. We provide a full description of the model architectures and hyperparameter choices for all four models applied to the full cow chromosome dataset (Supplementary Section 5). To determine when to stop training, we monitored the F1 score since it balances precision and recall. Training was terminated once F1 score no longer improved.

## Inference and Evaluation

We generated synthetic population under two scenarios. In the unconditional setting, the only required input was latent noise sampled from the Gaussian prior used during training. In the conditional setting, phenotype values were additionally sampled from the training set and provided as conditioning inputs. All metrics, except for the phenotype-prediction metric, were computed on the validation set. For the phenotype-prediction metric, we selected the

2 https://www.ukbiobank.ac.uk/

3 https://www.cog-genomics.org/plink/1.9/
4 https://faculty.washington.edu/browning/beagle/beagle.html
5 https://www.ensembl.org/index.html

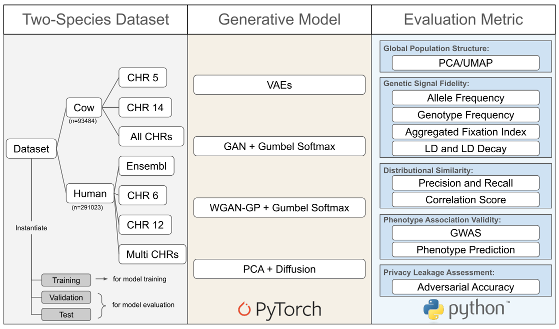


**Figure 1.** Schema of our generative modeling task. We used data from two species and constructed sub-datasets at different scales, ranging from single-chromosome to multi-chromosome settings. For human dataset, the Multi CHRs setting included chromosomes 3, 6, 12, and 17. Four generative models were implemented: VAE, GAN with Gumbel-Softmax, WGAN-GP with Gumbel-Softmax, and PCA with Diffusion Model. These models were evaluated across different aspects relevant to performance.

best model using the validation set and reported its performance on the test set. All metrics were averaged over 5 independent runs and 10,000 synthetic samples were generated per run. For metrics that require hyperparameter tuning, we suggest selecting the values that deliver satisfactory performance on two real datasets.

# Results

## Do Generative Models Capture the Statistical and Genetic Structure of the Real Population?

***Global Distribution Resemblance.*** A preliminary UMAP visualization of real and synthetic cow populations (Figure 2) shows that all models except GAN can approximate the overall data distribution. VAE and DM, both likelihood-based generative models, capture the global central structure well, with synthetic clusters centered around the real data. DM appears slightly better than VAE, as the latter shows more dispersion at the edges. WGAN performs best in this setting, effectively covering both the central structure and the broader population heterogeneity. To further investigate, we compared the first 32 principal components of WGAN-generated and real data and observed near-perfect alignment, suggesting strong distributional fidelity.

***Genetic Parameters and Linkage Structure Comparison.*** Figure 3 shows the comparison of allele and genotype frequencies between real and synthetic populations. Among all models, WGAN clearly outperforms the others, achieving perfect correlation score between real and synthetic frequency distributions. While the other models can partially capture the frequency profiles, we observed a consistent pattern of deviation: the frequency plots exhibit a sigmoid-like distortion. This phenomenon reflects a known Matthew effect [56, 57], where the model tends to overestimate high-frequency variants and underestimate rare ones, amplifying existing disparities in the data. For VAE, this phenomenon may be linked to its likelihood-based objective, which encourages prioritizing frequent patterns to maximize likelihood. DM performs better in this regard, possibly due to its hierarchical noise removal mechanism. GAN, known to suffer from mode collapse [33], exhibits this effect more severely. WGAN appears to be the only model that successfully mitigates this bias and accurately preserves the full frequency spectrum. Regarding LD, as shown in Figures 4, 5, and 6, all models except GAN manage to reproduce the original LD block structure and show a similar decay pattern with increasing distance. However, both VAE and WGAN tend to underestimate the strength of LD, while DM most closely matches the LD structure observed in the real population.

***Quantitative Evaluation Metrics.*** Table 1 summarizes the results across all quantitative metrics. For relatively small datasets (e.g., single chromosome in cow with around a thousand SNPs), VAE, WGAN, and DM perform well across most metrics. However, GAN suffers from mode collapse, leading to a recall score near 0. For larger-scale datasets (e.g., full chromosomes in cow and multiple chromosomes in human), WGAN consistently outperforms the other models. This is particularly evident for the recall metric: while all models tend to achieve high precision, WGAN is the only model that significantly improves recall. This aligns with the UMAP in Figure 2, which shows WGAN covering the full data distribution more effectively. Overall, WGAN achieves the best results across most metrics, although DM occasionally surpasses it in correlation score on human datasets.

***Factors Affecting the Complexity of Generative Modeling.*** Table 1 suggests that the difficulty of generative modeling is related to the input dimensionality: higher dimensions generally make learning more challenging, and we indeed observed this trend. However, we also observed that in the human dataset, CHR 6 has a higher input dimension than CHR 12, yet it is easier for models to learn. This indicates that input dimension alone does not fully explain the complexity. Upon further analysis, we found that SNP dependency also plays an important role. When SNPs exhibit stronger dependency, generative models can more easily capture the underlying distribution (Supplementary Section 6). Comparing across datasets, models consistently perform better on cow dataset than on human dataset. This may be attributed to differences in genetic structure: cow dataset exhibits stronger LD, implying higher SNP dependency, while human dataset shows greater genetic variability, which increases learning complexity.

***On the Robustness of Evaluation Metrics.*** When assessing the robustness of the evaluation metrics, we found that $F_{ST}^{aggregated}$, F1 and correlation score are highly correlated: good performance in one metric typically results in good performance across the others. A clear trade-off exists between precision and recall: models can achieve high precision by capturing only the core of the real distribution, whereas recall reflects how well the model covers the full diversity. A more detailed examination of the $AA$ score reveals an important nuance, consistent with findings reported in [23]: extreme scenarios may yield a favorable global $AA$ score while masking poor generative behavior. Ideally, both $AA_{real}$ and $AA_{syn}$ should be close to 0.50. However, when applying DM to human dataset, we observed a global $AA$ score of 0.50 resulting from an imbalanced case where $AA_{real} \approx 0$ and $AA_{syn} \approx 1$. This means that real samples are closer to synthetic ones than to other real samples, while synthetic samples are only close to each other and fail to reflect the diversity of the real distribution. This is also consistent with the observation that precision is close to 1 while recall is near 0, indicating that the model captures only a subset of the true distribution (Supplementary Section 7).

## Do Generative Models Preserve Geno–Pheno Association?

In the previous unconditional setting, WGAN and DM demonstrated superior performance, especially for large-dimensional datasets. We then evaluated their performance under the conditional setting by using phenotype as conditioning variable to investigate whether the models could also capture the genotype-phenotype association. Figure 7 presents the results of GWAS analysis on the full set of chromosomes in cow dataset, comparing real and synthetic populations. Both WGAN and DM are able to recover the 3 main quantitative trait locus (QTL) regions. When examining the regression coefficients $\beta$ in GWAS, we observed that the WGAN-generated synthetic popula-

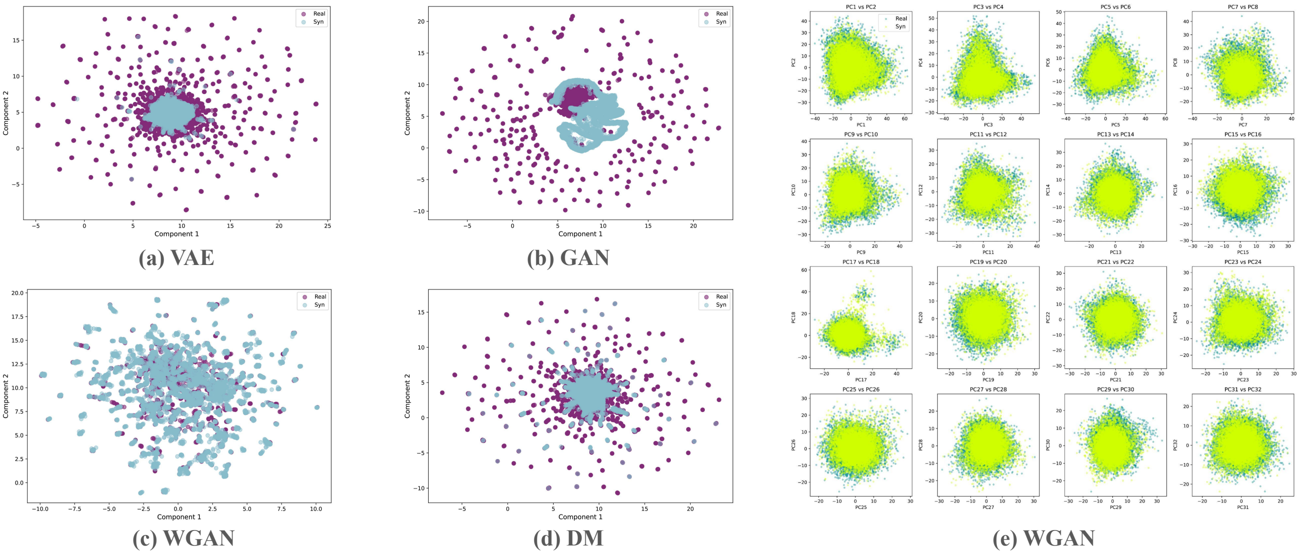


**Figure 2.** PCA and UMAP of real and model-generated synthetic populations on all chromosomes of cow dataset. (a), (b), (c), (d): UMAP of real and synthetic genotype generated by VAE, GAN, WGAN, and DM, respectively. (e): First 32 principal components of real and WGAN-generated synthetic genotype, explaining approximately 12% of the total variance.

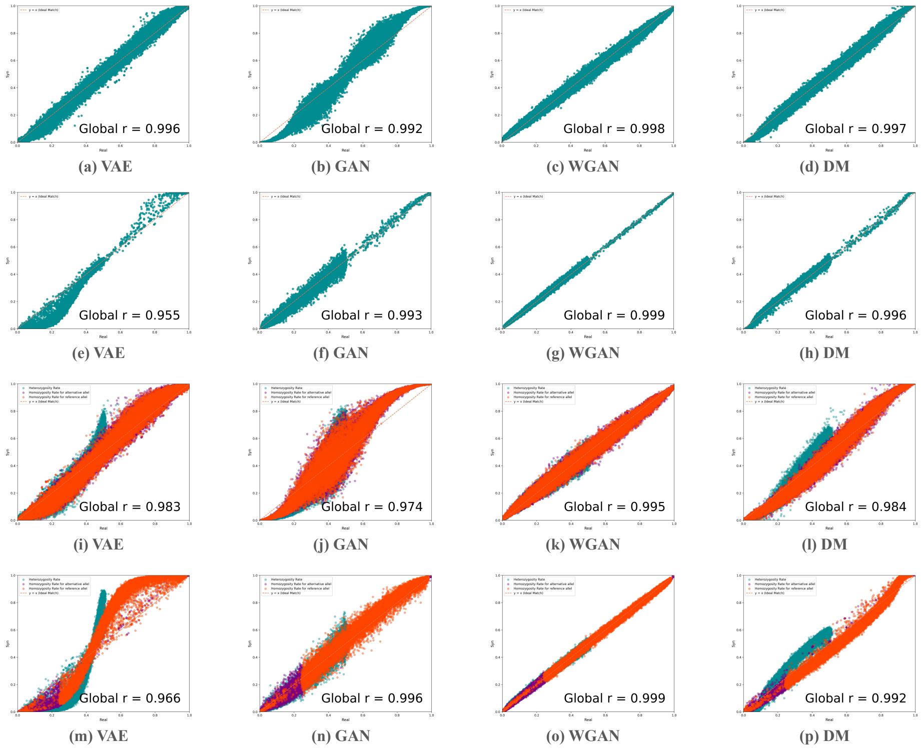


**Figure 3.** Comparison of genetic parameters between real and synthetic populations across all cow chromosomes and multiple human chromosomes. (a–d) Allele frequency comparison in cow. (e–h) Allele frequency comparison in human. (i–l) Genotype frequency comparison in cow. (m–p) Genotype frequency comparison in human.

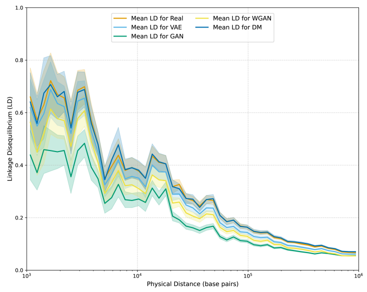


**Figure 4.** Comparison of linkage disequilibrium decay on cow chromosome 5.

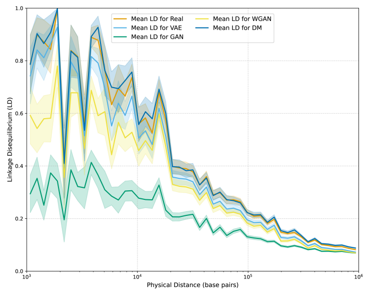


**Figure 5.** Comparison of linkage disequilibrium decay on cow chromosome 14.

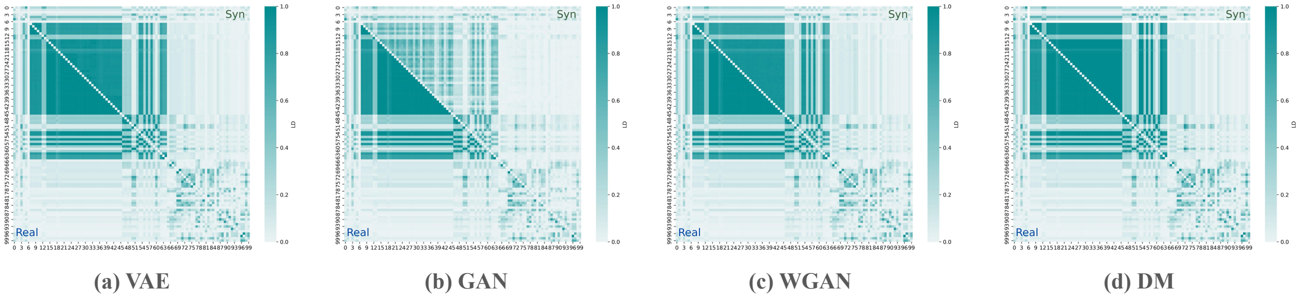


**Figure 6.** Comparison of linkage disequilibrium between real and synthetic cow populations on chromosome 14 using LD block heatmaps. The upper diagonal shows pairwise LD in the synthetic population, while the lower diagonal shows pairwise LD in the real population.

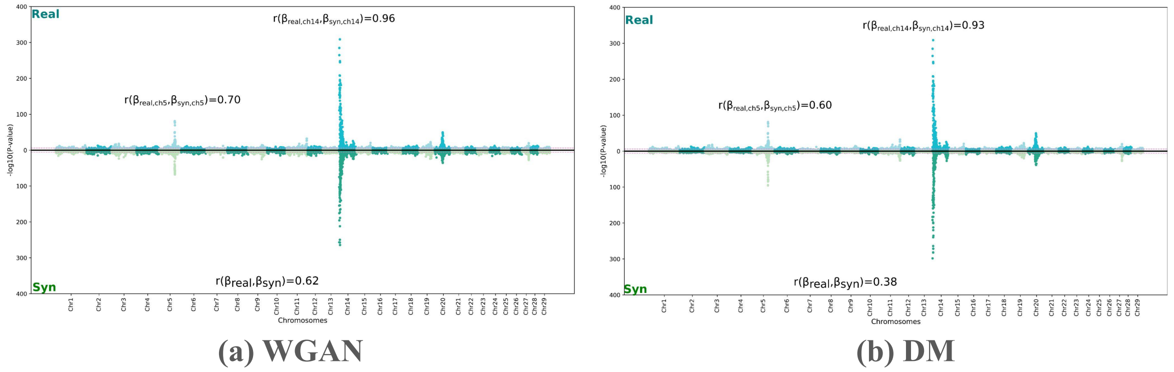


**Figure 7.** GWAS comparison of real and synthetic populations across all chromosomes of cow dataset. (a) Compared with WGAN-generated genotype. (b) Compared with DM-generated genotype.

**Table 1.** Quantitative performance indicators for all generative models on cow and human datasets.

| Dataset | Chromosome | Model | $F_{ST}^{aggregated}$ ↓ | Precision (%) ↑ | Recall (%) ↑ | F1 (%) ↑ | Corr(%) ↑ | AA |
|---|---|---|---|---|---|---|---|---|
| Cow | CHR 14 (1771 SNPs) | VAE | 1.81e−4 ± 7e−6 | 99.06 ± 0.04 | 99.70 ± 0.04 | 99.38 ± 0.02 | 96.87 ± 0.04 | 0.63 ± 2e−3 |
| | | GAN | 1.88e−4 ± 4e−6 | 80.88 ± 0.23 | 57.97 ± 0.45 | 67.53 ± 0.24 | 72.60 ± 0.13 | 0.99 ± 3e−4 |
| | | WGAN | **1.19e−4** ± 2e−6 | 99.64 ± 0.04 | **99.88 ± 0.02** | **99.76 ± 0.02** | **98.65 ± 0.02** | **0.55** ± 3e−3 |
| | | DM | 3.07e−4 ± 6e−6 | **99.92 ± 0.01** | 99.13 ± 0.03 | 99.52 ± 0.02 | 98.53 ± 0.01 | 0.63 ± 2e−3 |
| | CHR 5 (2238 SNPs) | VAE | 4.05e−4 ± 5e−6 | **99.87 ± 0.02** | 99.51 ± 0.03 | **99.69 ± 0.02** | 97.21 ± 0.04 | 0.68 ± 2e−3 |
| | | GAN | 3.99e−3 ± 4e−5 | 88.40 ± 0.21 | 0.01 ± 0.00 | 0.01 ± 0.01 | 55.40 ± 0.05 | 1.00 ± 7e−5 |
| | | WGAN | **1.22e−4** ± 4e−6 | 98.98 ± 0.07 | **99.86 ± 0.03** | 99.42 ± 0.04 | **98.74 ± 0.01** | **0.63** ± 2e−3 |
| | | DM | 3.10e−4 ± 4e−6 | 99.86 ± 0.02 | 99.26 ± 0.07 | 99.56 ± 0.04 | 98.17 ± 0.01 | 0.65 ± 3e−3 |
| | All CHRs (50161 SNPs) | VAE | 1.80e−3 ± 1e−5 | 99.99 ± 0.01 | 11.65 ± 1.03 | 20.85 ± 1.65 | 73.03 ± 0.11 | 0.96 ± 1e−3 |
| | | GAN | 5.58e−3 ± 1e−5 | **100 ± 0.00** | 0.00 ± 0.00 | 0.00 ± 0.00 | 0.52 ± 0.01 | 0.98 ± 2e−3 |
| | | WGAN | **6.21e−4** ± 5e−6 | 92.00 ± 0.16 | **99.93 ± 0.01** | **95.80 ± 0.09** | **83.32 ± 0.06** | **0.74** ± 7e−3 |
| | | DM | 1.10e−3 ± 1e−6 | **100 ± 0.00** | 40.59 ± 0.63 | 57.74 ± 0.64 | 76.56 ± 0.10 | 0.94 ± 1e−3 |
| Human | Ensembl (3493 SNPs) | VAE | 2.88e−2 ± 3e−5 | **100 ± 0.00** | 0.29 ± 0.24 | 0.57 ± 0.44 | 39.74 ± 1.35 | **0.50** ± 1e−5 |
| | | GAN | 5.00e−3 ± 9e−6 | 99.98 ± 0.01 | 0.00 ± 0.00 | 0.00 ± 0.00 | 34.03 ± 0.09 | 0.52 ± 5e−4 |
| | | WGAN | **1.31e−4** ± 2e−6 | 71.84 ± 0.11 | **97.86 ± 0.11** | **82.85 ± 0.11** | **83.74 ± 0.03** | 0.76 ± 1e−2 |
| | | DM | 1.53e−3 ± 5e−6 | **100 ± 0.00** | 13.96 ± 0.07 | 24.49 ± 0.11 | 61.73 ± 0.72 | **0.50** ± 3e−4 |
| | CHR 6 (12283 SNPs) | VAE | 6.08e−3 ± 3e−5 | 99.99 ± 0.01 | 0.05 ± 0.07 | 0.10 ± 0.13 | **64.93 ± 0.03** | **0.50** ± 6e−5 |
| | | GAN | 1.62e−3 ± 6e−6 | 99.02 ± 0.12 | 0.17 ± 0.06 | 0.34 ± 0.11 | 20.51 ± 0.46 | 0.52 ± 4e−4 |
| | | WGAN | **2.24e−4** ± 1e−6 | 57.76 ± 0.33 | **97.83 ± 0.11** | **72.63 ± 0.23** | 53.97 ± 0.10 | 0.73 ± 2e−2 |
| | | DM | 9.54e−4 ± 4e−6 | **100 ± 0.00** | 1.20 ± 0.05 | 2.36 ± 0.09 | 54.65 ± 0.77 | **0.50** ± 9e−5 |
| | CHR 12 (9780 SNPs) | VAE | 1.40e−2 ± 5e−5 | 99.99 ± 0.01 | 0.04 ± 0.01 | 0.08 ± 0.03 | 26.91 ± 0.21 | **0.50** ± 6e−4 |
| | | GAN | 9.20e−4 ± 6e−6 | 21.15 ± 0.25 | 0.95 ± 0.09 | 1.82 ± 0.17 | 8.30 ± 0.08 | 0.99 ± 6e−3 |
| | | WGAN | **1.13e−4** ± 1e−6 | 55.28 ± 0.72 | **75.19 ± 0.53** | **63.71 ± 0.57** | 40.16 ± 0.19 | 0.55 ± 4e−3 |
| | | DM | 9.23e−4 ± 3e−6 | **100 ± 0.00** | 1.20 ± 0.03 | 2.38 ± 0.06 | **40.35 ± 0.30** | **0.50** ± 1e−4 |
| | Multi CHRs (42409 SNPs) | VAE | 1.77e−2 ± 5e−5 | **100 ± 0.00** | 0.00 ± 0.00 | 0.00 ± 0.00 | 5.57 ± 0.07 | **0.50** ± 2e−3 |
| | | GAN | 1.46e−3 ± 5e−6 | **100 ± 0.01** | 0.65 ± 0.03 | 1.30 ± 0.05 | 5.10 ± 0.17 | 0.51 ± 7e−5 |
| | | WGAN | **1.63e−4** ± 1e−6 | 45.80 ± 0.42 | **64.35 ± 1.28** | **53.50 ± 0.46** | 19.58 ± 0.50 | 0.52 ± 1e−2 |
| | | DM | 9.56e−4 ± 3e−6 | **100 ± 0.00** | 1.00 ± 0.01 | 1.98 ± 0.02 | **20.04 ± 0.23** | **0.50** ± 8e−5 |

**Table 2.** Comparison of phenotype-prediction performance using real and synthetic genotype data.

| Dataset | | | XGBoost | | MLP | |
|---|---|---|---|---|---|---|
| | | | MSE ↓ | r ↑ | MSE ↓ | r ↑ |
| Cow | CHR 14 | Real | 0.60 ± 0.0054 | 0.65 ± 0.0034 | 0.67± 0.0088 | 0.61 ± 0.0066 |
| | | WGAN | 0.62 ± 0.0050 | 0.63 ± 0.0012 | **0.72 ± 0.0323** | **0.59 ± 0.0041** |
| | | DM | **0.61 ± 0.0056** | **0.64 ± 0.0023** | 0.74 ± 0.0188 | 0.55 ± 0.0033 |
| | CHR 5 | Real | 0.95 ± 0.0004 | 0.23 ± 0.0031 | 1.12 ± 0.0096 | 0.16 ± 0.0082 |
| | | WGAN | **0.95 ± 0.0014** | **0.23 ± 0.0021** | **1.14 ± 0.0168** | **0.15 ± 0.0151** |
| | | DM | 0.96 ± 0.0019 | 0.21 ± 0.0045 | 1.14 ± 0.0320 | 0.11± 0.0305 |
| | All CHRs | Real | 0.46 ± 0.0053 | 0.76 ± 0.0023 | 0.40 ± 0.0135 | 0.81 ± 0.0042 |
| | | WGAN | **0.52 ± 0.0069** | **0.72 ± 0.0027** | 0.49 ± 0.0261 | 0.75 ± 0.0067 |
| | | DM | 0.53 ± 0.0145 | 0.71 ± 0.0084 | **0.47 ± 0.0248** | **0.76 ± 0.0025** |
| Human | Ensembl | Real | 40.87± 0.1936 | 0.72 ± 0.0016 | 68.33 ± 6.1033 | 0.64 ± 0.0473 |
| | | WGAN | 43.44 ± 0.1392 | **0.70 ± 0.0011** | **69.87 ± 9.3012** | **0.59 ± 0.0105** |
| | | DM | **43.28 ± 0.6887** | **0.70 ± 0.0047** | 114.62 ± 31.7599 | 0.49± 0.0320 |
| | CHR 6 | Real | 42.81 ± 0.0791 | 0.71 ± 0.0006 | 88.53 ± 4.7244 | 0.44 ± 0.0384 |
| | | WGAN | **43.30 ± 0.1146** | **0.70 ± 0.0010** | **94.62 ± 5.2922** | **0.40 ± 0.0221** |
| | | DM | 44.65 ± 0.9555 | 0.69 ± 0.0079 | 218.95 ± 27.5067 | 0.24 ± 0.0099 |
| | CHR 12 | Real | 42.96 ± 0.0912 | 0.70 ± 0.0007 | 94.33 ± 5.6021 | 0.46 ± 0.0097 |
| | | WGAN | **43.41 ± 0.0684** | **0.70 ± 0.0006** | **96.52 ± 6.2831** | **0.42 ± 0.0158** |
| | | DM | 45.15 ± 1.4170 | 0.69 ± 0.0123 | 237.82 ± 14.8840 | 0.26 ± 0.0036 |
| | Multi CHRs | Real | 42.63 ± 0.1547 | 0.71 ± 0.0013 | 92.91 ± 3.2856 | 0.26 ± 0.0327 |
| | | WGAN | **43.44 ± 0.0800** | **0.70 ± 0.0006** | **96.51 ± 1.4907** | **0.23 ± 0.0334** |
| | | DM | 44.65 ± 0.9555 | 0.69 ± 0.0079 | 149.87 ± 5.7670 | 0.18 ± 0.0096 |

tion shows a higher correlation with the real population's β values compared to the DM-generated population.

Table 2 summarizes the predictive performance of machine learning and deep learning models on synthetic genotype to predict the conditioning phenotype, compared to the results from the real population. Similarly to the previous unconditional setting, for relatively small datasets (e.g., single chromosome in cow), both models achieve comparable performance to that obtained with real datasets. However, for more complex datasets, WGAN-generated synthetic genotype appears to better preserve the complex genotype-phenotype relationship, especially when using MLP as the prediction model. This aligns with WGAN's ability to improve the recall metric and more fully capture the distribution of real data. Overall, these results suggest that WGAN is able to generate synthetic population with genotype–phenotype association that closely mirror those observed in real data, as reflected by its consistently strong predictive performance across datasets and predictive model types.

## Discussion and Conclusion

The primary objective of this study was to investigate the effectiveness of widely used deep generative models for simulating genotype. We proposed specific adaptation for VAE, GAN, WGAN and diffusion models to better handle the discrete nature of genotype representation. Our experiments revealed that no single model performs best across all evaluation metrics and datasets. Each dataset exhibits distinct genetic properties, and we found that model performance is influenced by both the dimensionality of genotype sequence and the degree of SNP dependence. For relatively small and simple datasets (e.g., with a few thousand SNPs), we recommend using VAE due to its computational efficiency, training stability, and minimal hyperparameter tuning. For larger and more complex datasets with higher genetic diversity, WGAN-based model consistently outperforms the other models, particularly in capturing the overall distribution and the genotype-phenotype association.

We also proposed a comprehensive evaluation framework that combines multiple metrics to assess synthetic genotype quality from different angles. Since each metric captures a specific aspect, using them in combination provides a more complete evaluation. We found that not all previously developed metrics are robust. The *AA* score can produce misleading results in certain edge cases. Other metrics, such as the correlation score, are reliable but computationally intensive. Among all the metrics, recall stands out as a particularly valuable supervision signal during training, as it is more difficult to optimize and indicative of a model's ability to capture diversity. We recommend using PCA, $F_{ST}^{aggregated}$, precision, and recall during training to decide when to stop and only compute the more costly metrics afterward.

Our results are consistent with previous studies on haplotype generation, which have shown that generative models can accurately capture genetic structure. To our knowledge, this is the first work demonstrating that conditioning on phenotype enables generative models to generate synthetic populations that preserve genotype–phenotype associations. Such synthetic populations can be effectively applied to downstream tasks, such as GWAS, highlighting the potential of generative models to support genetics research. Furthermore, a trained generative model can function as a direct genotype–phenotype simulation tool, and the model itself can be

shared without releasing individual genomic sequences, thereby preserving privacy.

Several future research directions can be envisioned. In this study, we focused on models that learn the joint distribution of the entire genotype sequence, favoring a biologically grounded approach over sequential modeling. However, recent advances inspired by natural language processing, such as transformer-based models applied to DNA sequence [58–60], could also be adapted for genotype and merit further investigation. Another potential direction is the development of post-training refinement algorithms to improve the quality of generated sequences [61]. On the data side, future work could aim to better model additional features of genotype data, such as rare variants, population heterogeneity, and multi-phenotype conditioning. Incorporating modules that explicitly capture genotype–phenotype interaction could further enhance biological relevance. Lastly, exploring frugal learning strategies would be valuable, given the high dimensionality of genotype data and the computational demands of generative models.

## Availability of source code and requirements

The source code for the complete pipeline (model training, evaluation metrics, and experiments) is available on GitHub.

- **Project name:** DiscreteGenoGen
- **Project repository:** https://github.com/SihanXXX/DiscreteGenoGen
- **Operating system(s):** Platform independent
- **Programming language:** Python
- **Dependencies:** Listed in the `requirements.txt` file provided in the repository
- **License:** MIT License

## Data availability

Trained generative models for cows are available in our GitHub repository: https://github.com/SihanXXX/DiscreteGenoGen. These models can be directly used to simulate realistic genotype data and to reproduce our experiments. This supports the main practical motivation of our work: enabling genotype data sharing in a compact and privacy-preserving manner. A demonstration Jupyter Notebook for simulation is also provided at: https://github.com/SihanXXX/DiscreteGenoGen/tree/master/demo. Access to the UK Biobank dataset requires a separate application, which can be submitted at: https://www.ukbiobank.ac.uk/enable-your-research/apply-for-access.

## Abbreviations

VAE: Variational Autoencoder; DM: Diffusion Model; GAN: Generative Adversarial Network; SNP: Single Nucleotide Polymorphism; PCA: Principal Component Analysis; UMAP: Uniform Manifold Approximation and Projection; LD: Linkage Disequilibrium; ELBO: Evidence Lower Bound; GS: Gumbel–Softmax; WGAN: Wasserstein GAN; WGAN-GP: Wasserstein GAN with Gradient Penalty; GWAS: Genome-Wide Association Study; MLP: Multilayer Perceptron; AA: Nearest Neighbor Adversarial Accuracy; FST: Fixation Index; KNN: K-Nearest Neighbor; FC: Fat Content; YD: Yield Deviation; CHR: Chromosome;

## Additional Files

Supplementary material is available online.
**Supplementary Table 1.** Results of Principal Component Analysis (PCA) on genotype datasets.
**Supplementary Figure 1.** Illustration of a failed thresholding strategy when mapping generated continuous values to discrete genotypes.
**Supplementary Figure 2.** Exact genotype frequency matching using a thresholding strategy based on observed genotype frequencies.
**Supplementary Figure 3.** Precision and recall obtained with different distance metrics.
**Supplementary Figure 4.** Effect of SNP dependence on the difficulty of generative modeling.
**Supplementary Figure 5.** Detailed evaluation of the Nearest Neighbor Adversarial Accuracy (AA) score.
**Supplementary Figure 6.** Geometric illustration of a scenario where the AA score fails.

## Author Contributions

Data curation: SX, DB, TT. Conceptualization: SX, BH, JC, EB. Methodology: SX, BH, JC, EB. Formal analysis and investigation: SX. Writing—original draft: SX. Writing—review and editing: all authors. Supervision: BH, JC, EB.

## Funding

This work was supported by INRAE DigitBio Metaprogram.

## Acknowledgements

We thank Jocelyn De-Goër-De-Herve for managing the GPU infrastructure.

## Declarations

### Ethical Approval

Access to UK Biobank data was obtained under approved application 96326, and its use was conducted in accordance with all relevant guidelines and regulations. The acquisition of cow data was carried out in compliance with the ARRIVE guidelines.

### Consent for publication

Not applicable.

### Competing Interests

The authors declare that they have no competing interests.

## References

1. Reuter J, Spacek DV, Snyder M. High-Throughput Sequencing Technologies. Molecular Cell 2015;58(4):586–597.
2. Churko J, Mantalas G, Snyder M, et al. Overview of High Throughput Sequencing Technologies to Elucidate Molecular Pathways in Cardiovascular Diseases. Circulation research 2013 06;112:1613–23.
3. Gravel S. Population Genetics Models of Local Ancestry. Genetics 2012 06;191(2):607–619.
4. Kingman JFC. The coalescent. Stochastic Processes and their Applications 1982;13(3):235–248.
5. Hudson RR. Gene genealogies and the coalescent process. Oxford surveys in evolutionary biology 1990;7(1):44.
6. Kelleher J, Etheridge AM, McVean G. Efficient Coalescent Simu-

lation and Genealogical Analysis for Large Sample Sizes. PLOS Computational Biology 2016 05;12(5):1–22.
7. Hudson RR. Generating samples under a Wright–Fisher neutral model of genetic variation. Bioinformatics 2002 02;18(2):337–338.
8. Teshima KM, Innan H. mbs: modifying Hudson's ms software to generate samples of DNA sequences with a biallelic site under selection. BMC Bioinformatics 2009;10(1):166.
9. Baumdicker F, Bisschop G, Goldstein D, et al. Efficient ancestry and mutation simulation with msprime 1.0. Genetics 2021 12;220(3):iyab229.
10. Haller BC, Messer PW. SLiM 3: Forward Genetic Simulations Beyond the Wright–Fisher Model. Molecular Biology and Evolution 2019 01;36(3):632–637.
11. Ewing G, Hermisson J. MSMS: a coalescent simulation program including recombination, demographic structure and selection at a single locus. Bioinformatics 2010 06;26(16):2064–2065.
12. Peng B, Kimmel M. simuPOP: a forward-time population genetics simulation environment. Bioinformatics 2005 07;21(18):3686–3687.
13. Viñas R, Andrés-Terré H, Liò P, et al. Adversarial generation of gene expression data. Bioinformatics 2021 01;38(3):730–737.
14. Lacan A, Sebag M, Hanczar B. GAN-based data augmentation for transcriptomics: survey and comparative assessment. Bioinformatics 2023 06;39(Supplement_1):i111–i120.
15. Lacan A, André R, Sebag M, et al. In Silico Generation of Gene Expression profiles using Diffusion Models. bioRxiv 2024;.
16. Li Z, Ni Y, Huygelen TAB, et al., Latent Diffusion Model for DNA Sequence Generation; 2023.
17. Brixi G, Durrant MG, Ku J, et al. Genome modeling and design across all domains of life with Evo 2. bioRxiv 2025;.
18. Perera M, Montserrat DM, Barrabés M, et al. Generative Moment Matching Networks for Genotype Simulation. bioRxiv 2022;.
19. Geleta M, Montserrat DM, Giro-i Nieto X, et al. Deep Variational Autoencoders for Population Genetics. bioRxiv 2023;.
20. Montserrat DM, Bustamante C, Ioannidis A. Class-conditional vae-gan for local-ancestry simulation. arXiv preprint arXiv:191113220 2019;.
21. Nußberger J, Boesel F, Lenz S, et al. Synthetic observations from deep generative models and binary omics data with limited sample size. Briefings in Bioinformatics 2020 10;22(4):bbaa226.
22. Yelmen B, Decelle A, Ongaro L, et al. Creating artificial human genomes using generative neural networks. PLoS Genetics 2021 Feb;17(2):e1009303.
23. Yelmen B, Decelle A, Boulos LL, et al. Deep convolutional and conditional neural networks for large-scale genomic data generation. PLOS Computational Biology 2023;19(10):e1011584.
24. Szatkownik A, Furtlehner C, Charpiat G, et al. Latent generative modeling of long genetic sequences with GANs. bioRxiv 2024;p. 2024–08.
25. Szatkownik A, Planche L, Demeulle M, et al. Diffusion-based artificial genomes and their usefulness for local ancestry inference. bioRxiv 2024;p. 2024–10.
26. Kingma DP, Welling M. An Introduction to Variational Autoencoders. Foundations and Trends in Machine Learning 2019;12(4):307–392.
27. Goodfellow IJ, Pouget-Abadie J, Mirza M, et al. Generative Adversarial Nets. In: Advances in Neural Information Processing Systems, vol. 27; 2014. .
28. Ho J, Jain A, Abbeel P. Denoising Diffusion Probabilistic Models. In: Advances in Neural Information Processing Systems 33: Annual Conference on Neural Information Processing Systems 2020, NeurIPS 2020, December 6-12, 2020, virtual; 2020. .
29. Kynkäänniemi T, Karras T, Laine S, et al. Improved Precision and Recall Metric for Assessing Generative Models. In: Advances in Neural Information Processing Systems, vol. 32; 2019. .
30. Wold S, Esbensen K, Geladi P. Principal component analysis. Chemometrics and Intelligent Laboratory Systems 1987;2(1):37–52. Proceedings of the Multivariate Statistical Workshop for Geologists and Geochemists.
31. Jang E, Gu S, Poole B. Categorical Reparameterization with Gumbel-Softmax. In: 5th International Conference on Learning Representations, ICLR 2017, Toulon, France, April 24-26, 2017, Conference Track Proceedings; 2017. .
32. Kusner MJ, Hernández-Lobato JM. Gans for sequences of discrete elements with the gumbel-softmax distribution. arXiv preprint arXiv:161104051 2016;.
33. Bau D, Zhu J, Wulff J, et al. Seeing What a GAN Cannot Generate. In: 2019 IEEE/CVF International Conference on Computer Vision, ICCV 2019, Seoul, Korea (South), October 27 - November 2, 2019; 2019. p. 4501–4510.
34. Arjovsky M, Chintala S, Bottou L. Wasserstein Generative Adversarial Networks. In: Proceedings of the 34th International Conference on Machine Learning, vol. 70 of Proceedings of Machine Learning Research; 2017. p. 214–223.
35. Gulrajani I, Ahmed F, Arjovsky M, et al. Improved Training of Wasserstein GANs. In: Advances in Neural Information Processing Systems, vol. 30; 2017. .
36. Mirza M, Osindero S. Conditional Generative Adversarial Nets. CoRR 2014;.
37. McInnes L, Healy J, Saul N, et al. UMAP: Uniform Manifold Approximation and Projection. The Journal of Open Source Software 2018;3(29):861.
38. Laland KN, Uller T, Feldman MW, et al. The extended evolutionary synthesis: its structure, assumptions and predictions. Proceedings of the Royal Society B: Biological Sciences 2015;282(1813):20151019.
39. Wright S. THE GENETICAL STRUCTURE OF POPULATIONS. Annals of Eugenics 1949;15(1):323–354.
40. Weir BS, Cockerham CC. Estimating F-statistics for the analysis of population structure. Evolution 1984 Nov;38(6):1358–1370.
41. Slatkin M. Linkage Disequilibrium: Understanding the Evolutionary Past and Mapping the Medical Future. Nature Reviews Genetics 2008;9(6):477–485.
42. Rogers AR, Huff C. Linkage disequilibrium between loci with unknown phase. Genetics 2009 Jul;182(3):839–844.
43. Simonyan K, Zisserman A. Very Deep Convolutional Networks for Large-Scale Image Recognition. In: 3rd International Conference on Learning Representations, ICLR 2015, San Diego, CA, USA, May 7-9, 2015, Conference Track Proceedings; 2015. .
44. Uffelmann E, Huang QQ, Munung NS, et al. Genome-wide association studies. Nature Reviews Methods Primers 2021 aug;1(1):59.
45. Yale A, Dash S, Dutta R, et al. Generation and Evaluation of Privacy Preserving Synthetic Health Data. Neurocomputing 2020 Nov;416:244–255.
46. Sudlow C, Gallacher J, Allen N, et al. UK Biobank: an open access resource for identifying the causes of a wide range of complex diseases of middle and old age. PLoS Medicine 2015;12(3):e1001779.
47. Tribout T, Ducrocq V, Boichard D. HSSGBLUP: a Single-Step SNP BLUP genomic evaluation software adapted to large livestock populations. In: Proceedings of the 6th International Conference of Quantitative Genetics; 2020. p. 2–12.
48. Fernando RL, Cheng H, Golden BL, et al. Computational strategies for alternative single-step Bayesian regression models with large numbers of genotyped and non-genotyped animals. Genetics Selection Evolution 2016;48(1):96.
49. Littlejohn M, Tiplady K, Fink T, et al. Sequence-based Association Analysis Reveals an MGST1 eQTL with Pleiotropic Effects on Bovine Milk Composition. Scientific Reports 2016 04;6.
50. Winter A, Krämer W, Werner F, et al. Association of a lysine-232/alanine polymorphism in a bovine gene encoding acyl-

CoA:Diacylglycerol acyltransferase (DGAT1) with variation at a quantitative trait locus for milk fat content. Proceedings of the National Academy of Sciences of the United States of America 2002 08;99:9300–5.

51. Mullaney JM, Mills RE, Pittard WS, et al. Small insertions and deletions (INDELs) in human genomes. Human Molecular Genetics 2010 09;19(R2):R131–R136.
52. Lango Allen H, Estrada K, Lettre G, et al. Hundreds of variants clustered in genomic loci and biological pathways affect human height. Nature 2010;467(7317):832–838.
53. Anderson CA, Pettersson FH, Clarke GM, et al. Data quality control in genetic case-control association studies. Nature Protocols 2010;5(9):1564–1573.
54. Tahimic C, Wang Y, Bikle D. Anabolic effects of IGF-1 signaling on the skeleton. Frontiers in Endocrinology 2013 02;4:6.
55. He K, Zhang X, Ren S, et al. Deep Residual Learning for Image Recognition. In: Proceedings of 2016 IEEE Conference on Computer Vision and Pattern Recognition CVPR '16; 2016. p. 770–778.
56. Gao C, Huang K, Chen J, et al. Alleviating matthew effect of offline reinforcement learning in interactive recommendation. In: Proceedings of the 46th international ACM SIGIR conference on research and development in information retrieval; 2023. p. 238–248.
57. Ganev G, Oprisanu B, Cristofaro ED, Robin Hood and Matthew Effects: Differential Privacy Has Disparate Impact on Synthetic Data; 2022.
58. Ji Y, Zhou Z, Liu H, et al. DNABERT: pre-trained Bidirectional Encoder Representations from Transformers model for DNA-language in genome. Bioinformatics 2021 02;37(15):2112–2120.
59. Zhang D, Zhang W, Zhao Y, et al. DNAGPT: A Generalized Pre-trained Tool for Multiple DNA Sequence Analysis Tasks. bioRxiv 2024;.
60. Dalla-Torre H, Gonzalez L, Mendoza-Revilla J, et al. Nucleotide Transformer: building and evaluating robust foundation models for human genomics. Nature Methods 2025;22:287–297.
61. Li Z, Ni Y, Xia G, et al. Absorb & Escape: Overcoming Single Model Limitations in Generating Heterogeneous Genomic Sequences. In: Advances in Neural Information Processing Systems, vol. 37; 2024. p. 21949–21978.

Manuscript Click here to access/download;Manuscript;Reference PDF.pdf

Placeholder for OUP logo oup.pdf

*GigaScience*, 2025, 1–12

doi: xx.xxxx/xxxx
Manuscript in Preparation
Paper

PAPER

# Learning Inherent Genetic Patterns and Trait Associations with Deep Generative Models for Discrete Genotype Simulation

Sihan Xie[1,*], Thierry Tribout[1], Didier Boichard[1], Blaise Hanczar[2,†], Julien Chiquet[3,†] and Eric Barrey[1,†]

[1]Université Paris-Saclay, INRAE, AgroParisTech, GABI, 78350, Jouy-en-Josas, France and [2]Université Paris-Saclay, Univ Evry, IBISC, 91020, Evry-Courcouronnes, France and [3]Université Paris-Saclay, AgroParisTech, INRAE, UMR MIA Paris-Saclay, 91120, Palaiseau, France

[*] Corresponding author: sihan.xie@inrae.fr
[†] These authors contributed equally to this work.

## Abstract

**Background:** Deep generative models open new avenues for simulating realistic genomic data while preserving privacy and addressing data accessibility constraints. While previous studies have primarily focused on generating gene expression or haplotype data, this study explores generating genotype data in both unconditioned and phenotype-conditioned settings, which is inherently more challenging due to the discrete nature of genotype data.
**Results:** We developed and evaluated commonly used generative models, including Variational Autoencoders (VAEs), Diffusion Models, and Generative Adversarial Networks (GANs), and proposed adaptation tailored to discrete genotype data. We conducted extensive experiments on large-scale datasets, including all chromosomes from cow and multiple chromosomes from human. Model performance was assessed using a well-established set of metrics drawn from both deep learning and quantitative genetics literature. Our results show that these models can effectively capture genetic patterns and preserve genotype–phenotype association.
**Conclusions:** As deep generative models are able to reproduce key characteristics of genotype data, they can serve as direct tools for genotype–phenotype simulation, while also enabling privacy-preserving data sharing. Our findings provide a comprehensive evaluation of these models and offer practical guidance for future research in genotype simulation.

**Key words**: Deep Generative Models, Genotype Simulation, Quantitative Genetics

## Introduction

The development of dense genotyping platforms and high-throughput sequencing technologies has significantly advanced genetic analysis [1, 2]. Today, genomic studies rely on large biobanks that contain vast amounts of genomic data. However, working with such datasets presents several challenges, including high sequencing costs, substantial storage requirements, privacy concerns, and access restrictions that limit data sharing. To address these issues, simulation tools and synthetic data are commonly used. Traditional statistical simulation methods are based on evolutionary models like Wright-Fisher model [3] and coalescent theory [4–6], where users need to specify evolutionary parameters or provide ancestral population. While these simulation tools [7–12] are powerful, they often simplify various aspects of population genetics, which may not fully capture the complexities of real-world datasets.

Recently, data-driven simulation methods based on deep generative models have gained attention in genomics. These approaches eliminate the need to explicitly specify genetic parameters by learning directly from data, enabling the reproduction of fine-scale ge-

**Compiled on:** August 26, 2025.
Draft manuscript prepared by the author.

nomic characteristics presented in the given population. By shifting from explicit genomic sequences to generative models, the genome-wide data remains private, while the trained models can be shared publicly without directly exposing individual-level genetic information.

Previous studies have applied generative models to various genomic modalities: [13–15] focused on gene expression data, [16, 17] focused on DNA sequence, and there is a substantial body of literature on haplotype data [18–25]. In this work, we propose a new study on genotype data, which represents genetic variation at specific positions in the genome known as Single Nucleotide Polymorphisms (SNPs). Unlike binary-valued haplotype, genotype for diploid organisms includes three possible values (0, 1, 2), representing the number of alternative alleles inherited from both parents, which introduces specific modeling challenges. Importantly, directly simulating genotype data offers several advantages over haplotype-based approaches: Haplotype-based simulation methods lack rich conditioning capabilities, whereas our method allows conditioning on phenotypic traits, enabling more flexible and application-oriented use cases. Furthermore, traditional genotype–phenotype simulation workflows via haplotypes involve a multi-step pipeline and our approach consolidates this into a single generative step, reducing complexity and avoiding additional modeling assumptions. Finally, haplotype simulators are limited to genomic regions with strong linkage disequilibrium (LD) on a single chromosome, whereas our study supports genome-wide simulation, demonstrated in our experiments on cow dataset where we modeled genotypes across all 29 autosomes simultaneously, significantly extending the scale of genomic data simulation.

This paper investigates the use of deep generative models for simulating genotype data, potentially conditioned on phenotype. Specifically, we adapt models such as Variational Autoencoders (VAEs) [26], Generative Adversarial Networks (GANs) [27], and Diffusion Models [28] to accommodate the discrete nature of genotype. Properly evaluating synthetic genotype is a critical aspect of our study, as the evaluation metrics commonly used in the Generative AI community, such as precision and recall [29], have never been applied in previous haplotype generation studies. We propose a comprehensive evaluation framework that integrates both deep learning and quantitative genetics approaches, providing a rigorous comparison of the reviewed models. The paper first introduces the generative models adapted for genotype data, followed by a description of our proposed evaluation framework. We then detail the experimental setup and present the main results. Finally, we discuss how the proposed models can be practically implemented, along with potential challenges and future research directions.

## Generative Models for Genotype

Building on recent advances in haplotype generation [19, 22–25], we adopt generative models such as Variational Autoencoders (VAEs) [26], Generative Adversarial Networks (GANs) [27], and diffusion models [28]. They are well-suited for capturing global dependencies across all SNPs, as opposed to relying on sequential autoregressive decomposition, which may not align with the underlying biological structure. Since genotype is a discrete sequence represented as $\mathbf{x} \in \{0, 1, 2\}^n$, we propose adaptation to better handle this structure.

### Variational Autoencoders

Variational Autoencoders (VAEs) [26] learn to approximate the underlying data distribution by introducing a latent variable. A VAE consists of two neural networks, parameterized by $\phi$ and $\theta$: an encoder that maps the input data $x$ to a latent representation $z$ via the approximate posterior $q_\phi(z \mid x)$, and a decoder that reconstructs $x$ from $z$ via the likelihood $p_\theta(x \mid z)$. The model is trained by maximizing the Evidence Lower BOund (ELBO) on the marginal likelihood $\log p(x)$, using the reparameterization trick to enable efficient gradient-based optimization. The ELBO is given by

$$\mathcal{L}(\theta, \phi; x) = \underbrace{\mathbb{E}_{q_\phi(z|x)}\left[\log p_\theta(x \mid z)\right]}_{\text{decoder for reconstruction}} - \underbrace{D_{\mathrm{KL}}\left(q_\phi(z \mid x) \,\|\, p(z)\right)}_{\text{encoder for prior matching}}, \quad (1)$$

where $p(z)$ is the prior on the latent variable and $D_{\mathrm{KL}}$ denotes the Kullback-Leibler divergence. Optimizing this objective encourages the model to learn a meaningful, structured latent space that can be sampled to generate new, realistic data. Specifically, new samples are obtained by drawing a latent vector $z$ from the prior and passing it through the decoder.

### Diffusion Models

Diffusion models (DMs), and in particular Denoising Diffusion Probabilistic Models (DDPMs) [28], can be viewed as a Markovian hierarchical VAE where each latent $x_t$ has the same dimension as the data $x_0$ and the encoder is not learned but is a fixed Gaussian noising process. During the encoding phase, also called the forward diffusion process, we gradually add Gaussian noise to input $x_0$ until it becomes pure noise $x_T$ over $T$ steps via a Markov chain:

$$q(x_t \,|\, x_{t-1}) = \mathcal{N}(\sqrt{\alpha_t}\, x_{t-1},\ \beta_t \mathbf{I}), \quad \alpha_t = 1 - \beta_t \quad \text{for t} = 1, \ldots, T. \quad (2)$$

Because of the Markov property, the Gaussian transition, and the independence of the noise at every step, one can collapse all $t$ steps into a single closed-form marginal:

$$q(x_t \,|\, x_0) = \mathcal{N}\Big(x_t;\ \sqrt{\bar\alpha_t}\, x_0,\ (1 - \bar\alpha_t)\mathbf{I}\Big), \quad \bar\alpha_t = \prod_{s=1}^{t} \alpha_s. \quad (3)$$

Intuitively, the hyperparameter $\beta_t$ controls the amount of noise injected at step $t$, and $\alpha_t = 1 - \beta_t$ is the fraction of signal retained. Our goal is to undo the added noise by learning $p_\theta(x_{t-1} \mid x_t)$, so that starting from $x_T \sim \mathcal{N}(0, I)$ we can step-by-step recover $x_0$. The true reverse $q(x_{t-1} \,|\, x_t)$ is intractable, but during training we know $x_0$. Hence, we can write down the exact one-step posterior as a Gaussian distribution with a closed-form mean $\mu_t(x_t, x_0)$ and variance $\sigma_t^2$:

$$q(x_{t-1} \mid x_t, x_0) = \mathcal{N}\Big(\frac{\sqrt{\alpha_t}\,(1 - \bar\alpha_{t-1})}{1 - \bar\alpha_t}\, x_t + \frac{\beta_t \sqrt{\bar\alpha_{t-1}}}{1 - \bar\alpha_t}\, x_0,\ \frac{\beta_t\,(1 - \bar\alpha_{t-1})}{1 - \bar\alpha_t}\,\mathbf{I}\Big). \quad (4)$$

For the reverse process, we learn to approximate the posterior in Equation 4. For the variance part, many implementations simply set $\sigma_t^2 = \beta_t$, which has a negligible loss on quality. For the mean part, since $\mu_t(x_t, x_0)$ requires the true $x_0$ which is unavailable at inference, the direct training objective is to predict $x_0$ given $x_t$ and $t$. In practice, however, it is more common and empirically more stable to train a network $\epsilon_\theta(x_t, t)$ to predict the injected noise at each timestep optimized via mean-squared error loss. Then using the predicted noise $\epsilon_\theta(x_t, t)$, we first infer an estimator of $x_0$, given by $\hat{x}_0 = \left(x_t - \sqrt{1 - \bar\alpha_t}\ \epsilon_\theta(x_t, t)\right) / \sqrt{\bar\alpha_t}$. Substituting $\hat{x}_0$ for $x_0$ in Equation 4 gives the familiar reverse-step update:

$$x_{t-1} = \frac{1}{\sqrt{\alpha_t}}\Big(x_t - \frac{\beta_t}{\sqrt{1-\bar\alpha_t}}\, \epsilon_\theta(x_t, t)\Big) + \sigma_t z, \quad z \sim \mathcal{N}(0, I). \quad (5)$$

Despite their success, DMs are not compatible with discrete data. Two main strategies have been proposed to address this limitation: (1) defining a diffusion-like process that operates in discrete space,

or (2) projecting the discrete input into a continuous latent space. Additionally, DMs can be computationally demanding during inference. To address both issues, we adopt the second strategy. Various methods can be used to construct a suitable latent space. For example, [16] employed a VAE to embed DNA sequences into a continuous representation. We follow the PCA-based approach originally developed for haplotype [24, 25]. Specifically, we project genotype into a lower-dimensional PCA space [30] and train the DMs in this continuous latent space. This single transformation yields three major benefits in one shot: it greatly reduces dimensionality and speeds up both training and inference; it transforms the discrete genotype into a continuous representation that matches the assumptions of DMs; And it allows precise reconstruction via a simple linear multiplication (Supplementary Section 1).

### Generative Adversarial Networks

While VAEs and DMs learn the data distribution by explicitly maximizing likelihood, Generative Adversarial Networks (GANs) [27] adopt a fundamentally different strategy. They avoid explicit density estimation by framing generation as a two-player game: a generator $G$ transforms a latent vector $z \sim p_z$ (typically Gaussian) into a synthetic sample $G(z)$, and a discriminator $D$ attempts to distinguish real data from generated samples. Training proceeds by solving the minimax problem:

$$\min_G \max_D \underbrace{\mathbb{E}_{x\sim p_{\text{data}}}\left[\log D(x)\right] + \mathbb{E}_{z\sim p(z)}\left[\log(1-D(G(z)))\right]}_{\text{binary cross-entropy loss}}. \quad (6)$$

Here, $D$ is trained as a binary classifier to assign high probability to real sample $x$ and low probability to generated sample $G(z)$, while $G$ is trained to fool $D$ by producing ever more realistic outputs.

From Equation 6, $G$ is trained by backpropagating gradients from $D$ through its outputs $G(z)$. This works when $G(z)$ is continuous but fails for discrete outputs, which break differentiability. In previous GAN-based haplotype generation studies [21–23], no specific treatment was proposed for this issue: $G$ output continuous values between 0 and 1, which were passed directly to $D$ during training. At inference, discrete values were recovered using a binarization threshold of 0.5. This binary setting can be interpreted in two ways. First, from a probabilistic perspective, $G$ outputs the probability of class 1, and inference chooses the most likely class. Motivated by this view, we tested a probabilistic approach: using a Softmax final layer in $G$ to predict class probabilities per SNP, and training $D$ to distinguish these from one-hot encoded real genotype sequences. However, this method produced unsatisfactory results. The second interpretation views binarization as a quantization operation that maps continuous outputs to a discrete set. We therefore explored several threshold-based strategies for our ternary genotype data, but observed suboptimal results (Supplementary Section 2). To enable end-to-end differentiable training on discrete outputs, we instead integrate a Gumbel-Softmax [31, 32] layer into $G$. The Gumbel-Softmax distribution provides a continuous approximation to categorical sampling by applying a temperature-controlled softmax to perturbed logits. Concretely, for each SNP, if the final layer of $G$ produces logits $\ell = (\ell_0, \ell_1, \ell_2)$, we then sample Gumbel noise $g_i \sim \text{Gumbel}(0, 1)$, and compute the relaxed one-hot vector

$$\tilde{p}_i = \frac{\exp((\ell_i + g_i)/\tau)}{\sum_{j=0}^{2}\exp((\ell_j + g_j)/\tau)}, \quad (7)$$

where $\tau$ is a temperature parameter. As $\tau \to 0$, $\tilde{p}_i$ becomes exactly one-hot vector. During training we anneal $\tau$ from a high initial value down toward 0 to balance exploration and discretization. At inference we take $\arg\max_i \tilde{p}_i$ to recover a discrete value in $\{0, 1, 2\}$.

### Wasserstein GANs with Gradient Penalty

GANs lack an explicit likelihood measure and can suffer from training instabilities such as mode collapse [33]. Subsequent refinements like Wasserstein GAN (WGAN) [34] was developed to address these issues. The original WGAN replaces the Jensen-Shannon divergence with the Earth-Mover (Wasserstein-1) distance by solving:

$$\min_G \max_{D\in\mathcal{D}} \mathbb{E}_{x\sim p_{\text{data}}}\left[D(x)\right] - \mathbb{E}_{z\sim p_z}\left[D(G(z))\right], \quad (8)$$

where $\mathcal{D}$ is the set of 1-Lipschitz functions. While weight clipping was initially proposed to enforce the Lipschitz constraint, this approach is proved unstable in practice. The Wasserstein GAN with gradient penalty (WGAN-GP) [35] instead introduces an equivalent gradient penalty term that penalizes the deviation of the gradient norm from 1, leading to more stable training dynamics:

$$\min_G \max_D \underbrace{\mathbb{E}_{x\sim p_{\text{data}}}\left[D(x)\right] - \mathbb{E}_{z\sim p_z}\left[D(G(z))\right]}_{\text{Wasserstein distance between real and synthetic}} \underbrace{- \lambda\, \mathbb{E}_{\hat{x}\sim p_{\hat{x}}}\left(\|\nabla_{\hat{x}} D(\hat{x})\|_2 - 1\right)^2}_{\text{Gradient penalty}}, \quad (9)$$

where $p_{\hat{x}}$ is the distribution of points interpolated between real and generated samples, and $\lambda$ controls the penalty strength.

### Conditional Generative Modeling

So far, we have focused on modeling the marginal distribution $p(x)$. In practice, it is more interesting to learn the conditional distribution $p(x \mid y)$, which provides control over the generated data through conditioning variable $y$. A straightforward approach is to append $y$ to $x$ as input during training [36]. At inference, we can sample from $p(x \mid y)$ by specifying a desired value of $y$ to guide generation.

In previous work on haplotype generation [19, 20, 25], models have frequently used ancestry group as conditioning variable to reflect population structure. For genotype, a natural choice is phenotype, particularly quantitative traits, which are often associated with genetic variation and enable the generation of synthetic population that mirror specific trait distribution.

## Evaluation Metrics for Synthetic Genotype

Genotype lacks the intuitive visual or semantic cues of images and text. We therefore propose a diverse set of metrics that provides a multi-faceted assessment.

### PCA and UMAP visualization

The PCA [30] and UMAP [37] projections provide an initial visual assessment of how well the synthetic population resembles the real one. These dimensionality reduction techniques highlight global structure and potential clustering patterns, offering a qualitative sense of alignment between the two distributions. However, they do not quantitatively measure distributional similarity and should be interpreted as complementary to more rigorous evaluation metrics.

### Genetic Parameters

#### *Allele Frequency and Genotype Frequency*

We compare allele and genotype frequencies [38] between real and synthetic cohorts as a basic sanity check. Let $N$ be the num-

ber of individuals, for a given SNP $i$, let $n_{2,i}$, $n_{1,i}$, and $n_{0,i}$ denote the counts of individuals with genotype 2, 1, and 0, respectively. The allele frequency at locus $i$ is $p_i = \left(2n_{2,i} + n_{1,i}\right)/(2N)$. The genotype frequency is the proportion of each genotype class, given by $f_i(2) = n_{2,i}/N$(homozygous alternative), $f_i(1) = n_{1,i}/N$(heterozygous), $f_i(0) = n_{0,i}/N$(homozygous reference). A strong concordance indicates that the model has accurately reproduced the per-locus marginal distribution, which is a prerequisite before assessing the higher order structure.

### *Aggregated Fixation Index*

The fixation index $F_{ST}$ [39, 40] is a widely used population genetic statistic that quantifies the degree of genetic differentiation among populations. It normalizes the difference between the total heterozygosity and the average heterozygosity within populations, yielding a value between 0 (no genetic differentiation) and 1 (complete genetic differentiation). For SNP $i$, let $p_{\text{real},i}$ and $p_{\text{syn},i}$ denote the allele frequencies in the real and synthetic cohorts respectively, assuming both cohorts are of the same size. Thus, the combined allele frequency for the total population is $p_{T,i} = \left(p_{real,i} + p_{syn,i}\right)/2$. Recall that for a given SNP $i$, the expected heterozygosity is given by $H = 1 - p^2 - (1-p)^2$. Thus, for the two subpopulations we have $H_{real,i} = 1 - p_{real,i}^2 - (1 - p_{real,i})^2$ and $H_{syn,i} = 1 - p_{syn,i}^2 - (1 - p_{syn,i})^2$. The average within-subpopulation heterozygosity is $H_{S,i} = (H_{real,i} + H_{syn,i})/2$. The heterozygosity in the combined population is $H_{T,i} = 1 - p_{T,i}^2 - (1 - p_{T,i})^2$. The per-SNP fixation index is given by

$$F_{ST}(i) = \frac{H_{T,i} - H_{S,i}}{H_{T,i}}. \tag{10}$$

Recognizing that not all SNPs are equally informative, with those exhibiting higher heterozygosity providing greater insight into genetic diversity, we aggregate the per-SNP fixation index into a summary metric using a weighted average:

$$F_{ST}^{\text{aggregated}} = \frac{\sum_i H_{T,i} F_{ST}(i)}{\sum_i H_{T,i}} \tag{11}$$

### *Linkage Disequilibrium and Its Decay with Physical Distance Along Chromosome*

Linkage disequilibrium (LD) [41] measures the non-random association of alleles at different loci. Its decay with increasing physical distance along a chromosome reflects the effect of recombination in reshuffling genetic variation. For diploid genotype data, the unknown gametic phase complicates the accurate computation of LD statistics. To address this, we employ a fast estimator introduced in [42], which approximates LD between two loci without relying on the assumption of random mating or requiring iterative computation. This method is implemented in the *scikit-allel* Python library[1].

## Unsupervised Metrics for Structural Similarity

### *Precision and Recall*

Precision and recall, originally developed for classification, have been adapted to assess generative models [29]. Here, precision measures the quality of synthetic data by quantifying the fraction of generated samples that fall within the support of the real data distribution, while recall measures the diversity of synthetic data by quantifying the fraction of real samples that fall within the support of the synthetic data distribution. The F1 score is the harmonic mean of precision and recall.

To estimate the support of a data distribution, we define, for each sample in this dataset, a threshold $\epsilon$ as the distance to its $k^{th}$ nearest neighbor within the same set. This distance serves as the radius of a hypersphere centered on that sample, and the union of all such hyperspheres provides an estimate of the underlying manifold. Formally, let $R$ denote the set of real samples and $S$ the set of synthetic samples. Precision and recall are defined as follows:

$$\text{Precision} = \frac{1}{|S|}\sum_{s\in S} \mathbf{1}\left\{\exists\, r \in R \text{ such that } \|s - r\| < \epsilon_r\right\}, \tag{12}$$

$$\text{Recall} = \frac{1}{|R|}\sum_{r\in R} \mathbf{1}\left\{\exists\, s \in S \text{ such that } \|s - r\| < \epsilon_s\right\}. \tag{13}$$

In image-based applications, precision and recall are computed on high-level feature vectors extracted from pretrained VGG-16 classifier [43]. For genotype, no widely accepted pretrained network exists, we therefore use the original data directly for evaluation. For KNN-based manifold estimation, the $L_2$ distance is conventionally employed. Given that genotype is discrete, we experimented with both $L_1$ and $L_2$ distances and found no significant differences in the resulting metrics (Supplementary Section 3). We adopted $L_2$ distance for its greater computational efficiency. The choice of $k$ is crucial and we selected the value that yielded approximately 90% precision and recall on two real datasets.

### *Correlation Score*

To compare the moments of the real and synthetic distributions, correlation score is proposed in [13], the idea is to compute the Pearson correlation coefficient between the strictly upper-diagonal elements of the SNP-pairwise correlation matrices $M_{real}$ and $M_{syn}$:

$$\rho(M_{\text{real}}, M_{\text{syn}}) = \frac{2}{n(n-1)}\sum_{i=1}^{n-1}\sum_{j=i+1}^{n} \frac{M_{i,j;\text{real}} - \mu(M_{\text{real}})}{\sigma(M_{\text{real}})} \times \frac{M_{i,j;\text{syn}} - \mu(M_{\text{syn}})}{\sigma(M_{\text{syn}})}, \tag{14}$$

where $n$ is the number of SNPs, $\mu(M)$ is the mean and $\sigma(M)$ is the standard deviation of the strictly upper-diagonal elements.

## Supervised Metrics for Geno–Pheno Association

### *Genome-wide association study (GWAS)*

In quantitative genetics, Genome-Wide Association Study (GWAS) [44] is a fundamental tool for identifying genetic variants associated with specific traits. In GWAS, a per-SNP regression is performed and a two-sided t-test is used to determine whether the regression coefficient $\beta$ is significantly different from 0. The corresponding $p$-value gives us the significance of the association. GWAS can be viewed as a feature-importance method, since each SNP's estimated effect size $\beta$ and its $p$-value indicate how strongly that locus contributes to phenotype prediction. By comparing GWAS results obtained from synthetic population with those from real, we can directly evaluate whether our generative model has preserved key biological signals.

### *Phenotype Prediction Performance*

We further assess synthetic genotype by evaluating its utility in phenotype prediction. Specifically, we train an XGBoost and a multilayer perceptron (MLP) on synthetic data, then assess their performance on an independent real dataset not used during generative training. If a predictive model trained solely on synthetic data performs comparably to one trained on real data, it suggests that the synthetic population has faithfully preserved the underlying genotype–phenotype relationship.

1 https://scikit-allel.readthedocs.io/

## Privacy Leakage Assessment

***Nearest Neighbor Adversarial Accuracy (AA)***

Since genotype data is highly sensitive, our synthetic data must balance utility with privacy protection. We adopt the nearest neighbour adversarial accuracy ($AA$) proposed in [45], which is conceptually similar to precision and recall. Rather than estimating the entire manifold with a full KNN approach, we use 1NN to compare local neighborhood distances. The intuition is that synthetic data should be close enough to real data to preserve utility, yet not so close as to risk privacy leakage. For each real sample, we measure whether its distance to its nearest synthetic neighbour ($d_{RS}$) is larger than its distance to its nearest real neighbour ($d_{RR}$). Likewise, for each synthetic sample, we check whether its distance to its nearest real neighbour ($d_{SR}$) is larger than its distance to its nearest synthetic neighbour ($d_{SS}$). These comparisons yield two values: one for the real dataset ($AA_{real}$) and one for the synthetic dataset ($AA_{syn}$). The overall $AA$ score is defined as the average of these two quantities. Formally, we have:

$$AA = \frac{1}{2}\Bigg( \underbrace{\frac{1}{N}\sum_{i=1}^{N} \mathbf{1}(d_{RS}(i) > d_{RR}(i))}_{AA_{\text{real}}} + \underbrace{\frac{1}{N}\sum_{i=1}^{N} \mathbf{1}(d_{SR}(i) > d_{SS}(i))}_{AA_{\text{syn}}} \Bigg). \tag{15}$$

Same as in the calculation of precision and recall, we use $L_2$ distance. An $AA$ value near 0 indicates overfitting, while an $AA$ value near 1 suggests underfitting. Ideally, an $AA$ value around 0.5 reflects a good tradeoff between utility and privacy.

# Experimental Setting

The following section describes our experimental setup, including datasets, model architectures, hyperparameter choices, synthetic data simulation, and metric computation. A schematic overview is provided in Figure 1.

## Datasets

Since SNP frequency distributions and correlations vary across populations, techniques developed in one group may not generalize well to others. Therefore, we used two large-scale datasets from different species: a Holstein cow cohort and the human dataset from UK Biobank[2] [46]. For diploid organisms, genotypes were encoded as 0 (homozygous reference), 1 (heterozygous), and 2 (homozygous alternate).

**Cow:** Our cow dataset comprises 93,484 individuals genotyped at 50,161 SNPs across all 29 pairs of autosomes. The selected phenotype is fat content (FC), a milk production trait that reflects the proportion of fat in milk. Fat is a key component in dairy products and influences the taste, texture, and richness of milk. FC has relatively high heritability, estimated at approximately 0.50. For selection purpose, FC was analyzed with a mixed model that accounts for various fixed environmental effects, the permanent environmental effect of the cow, and the breeding value (Supplementary Section 4). The so-called Yield Deviations (YD) are therefore by-products of the French Holstein Single Step genomic evaluation [47, 48]. The YD of FC for a cow is the mean of its phenotypes that have been adjusted for all non-genetic effects estimated in the genetic evaluation, and serves as the conditioning phenotype in our study. We assessed model performance on two individual chromosomes: Chromosome 5 (2,238 SNPs), where the MGST1 gene [49] is located, and Chromosome 14 (1,771 SNPs), where the DGAT1 gene [50] is located. In our experiments, these chromosomes exhibited the strongest GWAS signals for the selected trait. We also evaluated the models using the full concatenated genotype across all chromosomes.

**Human:** UK Biobank provides genotype and phenotype data for 488,377 participants, including 805,426 variants comprising both SNPs and INDELs [51], across the 22 autosomes, sex chromosomes, and the mitochondrial chromosome. We used sex and height as conditioning phenotypes, as height is a highly heritable and polygenic trait [52]. Following the pipeline proposed in [53] to assemble our study subsets, we performed quality control using PLINK 1.9[3], including checks for sex discordance, individual and SNP missingness, minor-allele-frequency filtering, Hardy–Weinberg equilibrium testing, and LD-based tag SNP selection. Missing genotypes were imputed using Beagle 5.4[4]. To recover biologically relevant height loci, we incorporated annotations from Ensembl[5] and extracted 3,493 SNPs associated with height. In a final cohort of 291,023 individuals, we constructed 4 genotype datasets: the 3,493 height-associated variants; Chromosome 6 (12,283 SNPs) where a QTL was detected by GWAS; Chromosome 12 (9,780 SNPs) where IGF-1 gene [54] is located; a combined set of 42,409 SNPs from Chromosomes 3, 6, 12, and 17.

For VAE, GAN, and WGAN models, genotype was first transformed using one-hot encoding. For DM, we applied PCA and retained the number of principal components that captured 90% of the total variance in each dataset. Across all experiments, 70% of samples were used for training, 15% for validation, and 15% for testing.

## Models and Training

All models were implemented with fully connected layers. Because genotype sequences lack inherent spatial or temporal structure, we avoided convolutional and recurrent layers. Each model consisted of a sequence of dense layers, with layer widths heuristically scaled to the data dimension. To improve training stability and gradient flow, we incorporated residual connections [55]. The VAE employed a symmetric encoder–decoder architecture. The GAN and WGAN shared identical network architectures. In WGAN, the gradient penalty coefficient $\lambda$ was set to 10, with 5 discriminator updates per generator update. For DM, we tested several noise schedules and found that a linear $\beta$ schedule gave the best performance.

We performed a grid search over network architecture and key training hyperparameters. We provide a full description of the model architectures and hyperparameter choices for all four models applied to the full cow chromosome dataset (Supplementary Section 5). To determine when to stop training, we monitored the F1 score since it balances precision and recall. Training was terminated once F1 score no longer improved.

## Inference and Evaluation

We generated synthetic population under two scenarios. In the unconditional setting, the only required input was latent noise sampled from the Gaussian prior used during training. In the conditional setting, phenotype values were additionally sampled from the training set and provided as conditioning inputs. All metrics, except for the phenotype-prediction metric, were computed on the validation set. For the phenotype-prediction metric, we selected the

2 https://www.ukbiobank.ac.uk/

3 https://www.cog-genomics.org/plink/1.9/

4 https://faculty.washington.edu/browning/beagle/beagle.html

5 https://www.ensembl.org/index.html

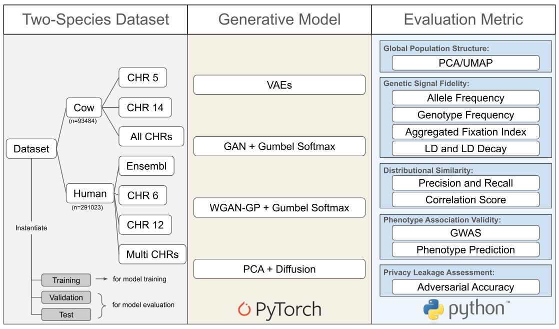


**Figure 1.** Schema of our generative modeling task. We used data from two species and constructed sub-datasets at different scales, ranging from single-chromosome to multi-chromosome settings. For human dataset, the Multi CHRs setting included chromosomes 3, 6, 12, and 17. Four generative models were implemented: VAE, GAN with Gumbel-Softmax, WGAN-GP with Gumbel-Softmax, and PCA with Diffusion Model. These models were evaluated across different aspects relevant to performance.

best model using the validation set and reported its performance on the test set. All metrics were averaged over 5 independent runs and 10,000 synthetic samples were generated per run. For metrics that require hyperparameter tuning, we suggest selecting the values that deliver satisfactory performance on two real datasets.

## Results

### Do Generative Models Capture the Statistical and Genetic Structure of the Real Population?

***Global Distribution Resemblance.*** A preliminary UMAP visualization of real and synthetic cow populations (Figure 2) shows that all models except GAN can approximate the overall data distribution. VAE and DM, both likelihood-based generative models, capture the global central structure well, with synthetic clusters centered around the real data. DM appears slightly better than VAE, as the latter shows more dispersion at the edges. WGAN performs best in this setting, effectively covering both the central structure and the broader population heterogeneity. To further investigate, we compared the first 32 principal components of WGAN-generated and real data and observed near-perfect alignment, suggesting strong distributional fidelity.

***Genetic Parameters and Linkage Structure Comparison.*** Figure 3 shows the comparison of allele and genotype frequencies between real and synthetic populations. Among all models, WGAN clearly outperforms the others, achieving perfect correlation score between real and synthetic frequency distributions. While the other models can partially capture the frequency profiles, we observed a consistent pattern of deviation: the frequency plots exhibit a sigmoid-like distortion. This phenomenon reflects a known Matthew effect [56, 57], where the model tends to overestimate high-frequency variants and underestimate rare ones, amplifying existing disparities in the data. For VAE, this phenomenon may be linked to its likelihood-based objective, which encourages prioritizing frequent patterns to maximize likelihood. DM performs better in this regard, possibly due to its hierarchical noise removal mechanism. GAN, known to suffer from mode collapse [33], exhibits this effect more severely. WGAN appears to be the only model that successfully mitigates this bias and accurately preserves the full frequency spectrum. Regarding LD, as shown in Figures 4, 5, and 6, all models except GAN manage to reproduce the original LD block structure and show a similar decay pattern with increasing distance. However, both VAE and WGAN tend to underestimate the strength of LD, while DM most closely matches the LD structure observed in the real population.

***Quantitative Evaluation Metrics.*** Table 1 summarizes the results across all quantitative metrics. For relatively small datasets (e.g., single chromosome in cow with around a thousand SNPs), VAE, WGAN, and DM perform well across most metrics. However, GAN suffers from mode collapse, leading to a recall score near 0. For larger-scale datasets (e.g., full chromosomes in cow and multiple chromosomes in human), WGAN consistently outperforms the other models. This is particularly evident for the recall metric: while all models tend to achieve high precision, WGAN is the only model that significantly improves recall. This aligns with the UMAP in Figure 2, which shows WGAN covering the full data distribution more effectively. Overall, WGAN achieves the best results across most metrics, although DM occasionally surpasses it in correlation score on human datasets.

***Factors Affecting the Complexity of Generative Modeling.*** Table 1 suggests that the difficulty of generative modeling is related to the input dimensionality: higher dimensions generally make learning more challenging, and we indeed observed this trend. However, we also observed that in the human dataset, CHR 6 has a higher input dimension than CHR 12, yet it is easier for models to learn. This indicates that input dimension alone does not fully explain the complexity. Upon further analysis, we found that SNP dependency also plays an important role. When SNPs exhibit stronger dependency, generative models can more easily capture the underlying distribution (Supplementary Section 6). Comparing across datasets, models consistently perform better on cow dataset than on human dataset. This may be attributed to differences in genetic structure: cow dataset exhibits stronger LD, implying higher SNP dependency, while human dataset shows greater genetic variability, which increases learning complexity.

***On the Robustness of Evaluation Metrics.*** When assessing the robustness of the evaluation metrics, we found that $F_{ST}^{aggregated}$, F1 and correlation score are highly correlated: good performance in one metric typically results in good performance across the others. A clear trade-off exists between precision and recall: models can achieve high precision by capturing only the core of the real distribution, whereas recall reflects how well the model covers the full diversity. A more detailed examination of the *AA* score reveals an important nuance, consistent with findings reported in [23]: extreme scenarios may yield a favorable global *AA* score while masking poor generative behavior. Ideally, both $AA_{real}$ and $AA_{syn}$ should be close to 0.50. However, when applying DM to human dataset, we observed a global *AA* score of 0.50 resulting from an imbalanced case where $AA_{real} \approx 0$ and $AA_{syn} \approx 1$. This means that real samples are closer to synthetic ones than to other real samples, while synthetic samples are only close to each other and fail to reflect the diversity of the real distribution. This is also consistent with the observation that precision is close to 1 while recall is near 0, indicating that the model captures only a subset of the true distribution (Supplementary Section 7).

### Do Generative Models Preserve Geno–Pheno Association?

In the previous unconditional setting, WGAN and DM demonstrated superior performance, especially for large-dimensional datasets. We then evaluated their performance under the conditional setting by using phenotype as conditioning variable to investigate whether the models could also capture the genotype-phenotype association. Figure 7 presents the results of GWAS analysis on the full set of chromosomes in cow dataset, comparing real and synthetic populations. Both WGAN and DM are able to recover the 3 main quantitative trait locus (QTL) regions. When examining the regression coefficients $\beta$ in GWAS, we observed that the WGAN-generated synthetic popula-

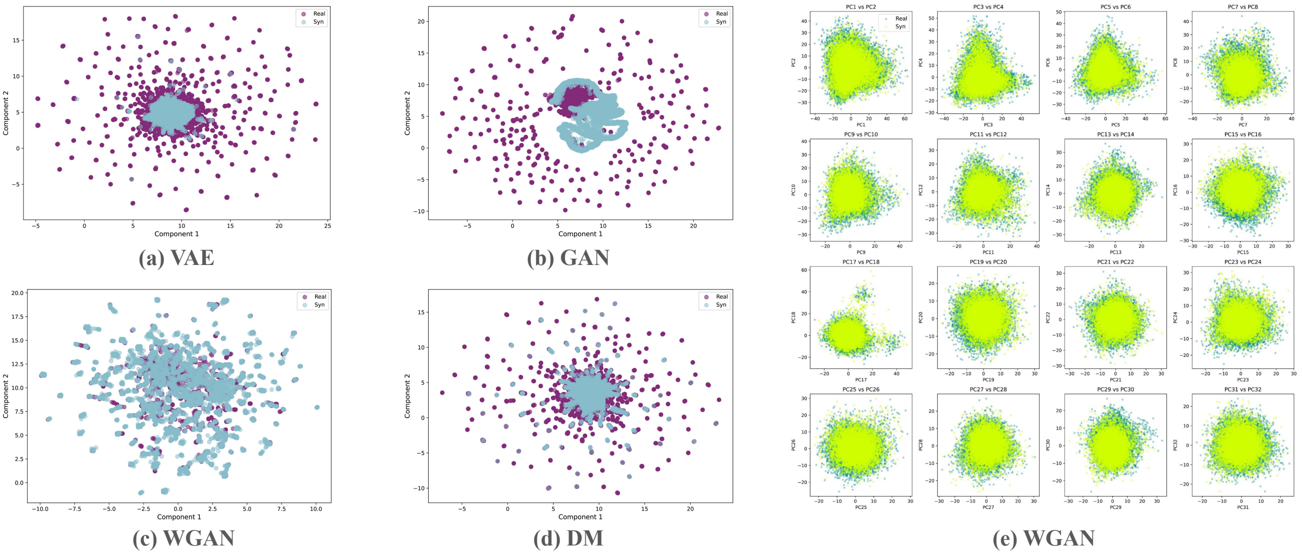


**Figure 2.** PCA and UMAP of real and model-generated synthetic populations on all chromosomes of cow dataset. (a), (b), (c), (d): UMAP of real and synthetic genotype generated by VAE, GAN, WGAN, and DM, respectively. (e): First 32 principal components of real and WGAN-generated synthetic genotype, explaining approximately 12% of the total variance.

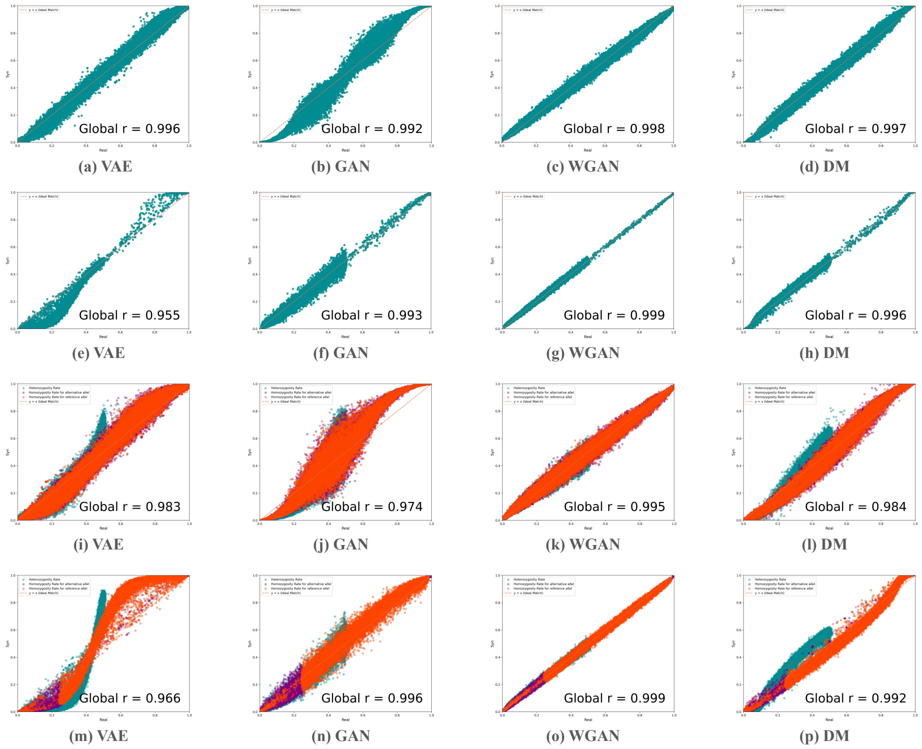


**Figure 3.** Comparison of genetic parameters between real and synthetic populations across all cow chromosomes and multiple human chromosomes. (a–d) Allele frequency comparison in cow. (e–h) Allele frequency comparison in human. (i–l) Genotype frequency comparison in cow. (m–p) Genotype frequency comparison in human.

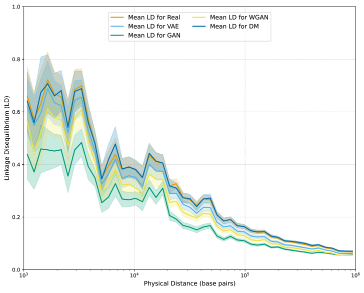


**Figure 4.** Comparison of linkage disequilibrium decay on cow chromosome 5.

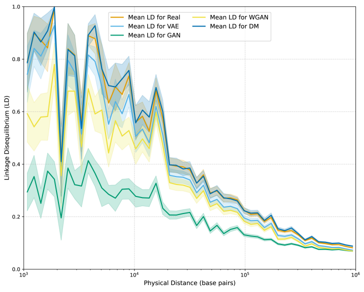


**Figure 5.** Comparison of linkage disequilibrium decay on cow chromosome 14.

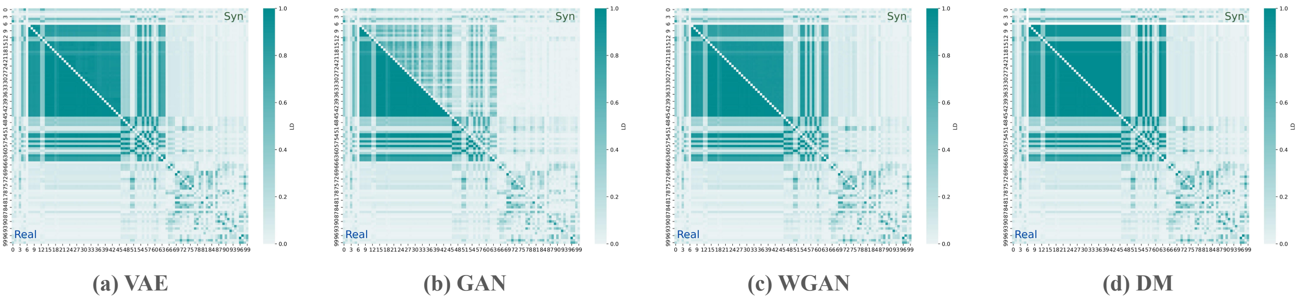


**Figure 6.** Comparison of linkage disequilibrium between real and synthetic cow populations on chromosome 14 using LD block heatmaps. The upper diagonal shows pairwise LD in the synthetic population, while the lower diagonal shows pairwise LD in the real population.

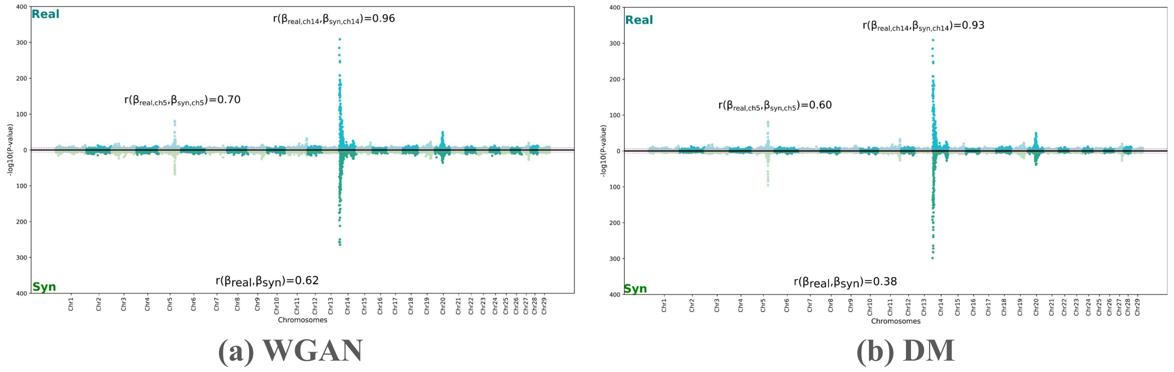


**Figure 7.** GWAS comparison of real and synthetic populations across all chromosomes of cow dataset. (a) Compared with WGAN-generated genotype. (b) Compared with DM-generated genotype.

**Table 1.** Quantitative performance indicators for all generative models on cow and human datasets.

| Dataset | Chromosome | Model | $F_{ST}^{aggregated}$ ↓ | Precision (%) ↑ | Recall (%) ↑ | F1 (%) ↑ | Corr(%) ↑ | AA |
|---|---|---|---|---|---|---|---|---|
| Cow | CHR 14 (1771 SNPs) | VAE | 1.81e−4 ± 7e−6 | 99.06 ± 0.04 | 99.70 ± 0.04 | 99.38 ± 0.02 | 96.87 ± 0.04 | 0.63 ± 2e−3 |
| | | GAN | 1.88e−4 ± 4e−6 | 80.88 ± 0.23 | 57.97 ± 0.45 | 67.53 ± 0.24 | 72.60 ± 0.13 | 0.99 ± 3e−4 |
| | | WGAN | **1.19e−4** ± 2e−6 | 99.64 ± 0.04 | **99.88 ± 0.02** | **99.76 ± 0.02** | **98.65 ± 0.02** | **0.55** ± 3e−3 |
| | | DM | 3.07e−4 ± 6e−6 | **99.92 ± 0.01** | 99.13 ± 0.03 | 99.52 ± 0.02 | 98.53 ± 0.01 | 0.63 ± 2e−3 |
| | CHR 5 (2238 SNPs) | VAE | 4.05e−4 ± 5e−6 | **99.87 ± 0.02** | 99.51 ± 0.03 | **99.69 ± 0.02** | 97.21 ± 0.04 | 0.68 ± 2e−3 |
| | | GAN | 3.99e−3 ± 4e−5 | 88.40 ± 0.21 | 0.01 ± 0.00 | 0.01 ± 0.01 | 55.40 ± 0.05 | 1.00 ± 7e−5 |
| | | WGAN | **1.22e−4** ± 4e−6 | 98.98 ± 0.07 | **99.86 ± 0.03** | 99.42 ± 0.04 | **98.74 ± 0.01** | **0.63** ± 2e−3 |
| | | DM | 3.10e−4 ± 4e−6 | 99.86 ± 0.02 | 99.26 ± 0.07 | 99.56 ± 0.04 | 98.17 ± 0.01 | 0.65 ± 3e−3 |
| | All CHRs (50161 SNPs) | VAE | 1.80e−3 ± 1e−5 | 99.99 ± 0.01 | 11.65 ± 1.03 | 20.85 ± 1.65 | 73.03 ± 0.11 | 0.96 ± 1e−3 |
| | | GAN | 5.58e−3 ± 1e−5 | **100 ± 0.00** | 0.00 ± 0.00 | 0.00 ± 0.00 | 0.52 ± 0.01 | 0.98 ± 2e−3 |
| | | WGAN | **6.21e−4** ± 5e−6 | 92.00 ± 0.16 | **99.93 ± 0.01** | **95.80 ± 0.09** | **83.32 ± 0.06** | **0.74** ± 7e−3 |
| | | DM | 1.10e−3 ± 1e−6 | **100 ± 0.00** | 40.59 ± 0.63 | 57.74 ± 0.64 | 76.56 ± 0.10 | 0.94 ± 1e−3 |
| Human | Ensembl (3493 SNPs) | VAE | 2.88e−2 ± 3e−5 | **100 ± 0.00** | 0.29 ± 0.24 | 0.57 ± 0.44 | 39.74 ± 1.35 | **0.50** ± 1e−5 |
| | | GAN | 5.00e−3 ± 9e−6 | 99.98 ± 0.01 | 0.00 ± 0.00 | 0.00 ± 0.00 | 34.03 ± 0.09 | 0.52 ± 5e−4 |
| | | WGAN | **1.31e−4** ± 2e−6 | 71.84 ± 0.11 | **97.86 ± 0.11** | **82.85 ± 0.11** | **83.74 ± 0.03** | 0.76 ± 1e−2 |
| | | DM | 1.53e−3 ± 5e−6 | **100 ± 0.00** | 13.96 ± 0.07 | 24.49 ± 0.11 | 61.73 ± 0.72 | **0.50** ± 3e−4 |
| | CHR 6 (12283 SNPs) | VAE | 6.08e−3 ± 3e−5 | 99.99 ± 0.01 | 0.05 ± 0.07 | 0.10 ± 0.13 | **64.93 ± 0.03** | **0.50** ± 6e−5 |
| | | GAN | 1.62e−3 ± 6e−6 | 99.02 ± 0.12 | 0.17 ± 0.06 | 0.34 ± 0.11 | 20.51 ± 0.46 | 0.52 ± 4e−4 |
| | | WGAN | **2.24e−4** ± 1e−6 | 57.76 ± 0.33 | **97.83 ± 0.11** | **72.63 ± 0.23** | 53.97 ± 0.10 | 0.73 ± 2e−2 |
| | | DM | 9.54e−4 ± 4e−6 | **100 ± 0.00** | 1.20 ± 0.05 | 2.36 ± 0.09 | 54.65 ± 0.77 | **0.50** ± 9e−5 |
| | CHR 12 (9780 SNPs) | VAE | 1.40e−2 ± 5e−5 | 99.99 ± 0.01 | 0.04 ± 0.01 | 0.08 ± 0.03 | 26.91 ± 0.21 | **0.50** ± 6e−4 |
| | | GAN | 9.20e−4 ± 6e−6 | 21.15 ± 0.25 | 0.95 ± 0.09 | 1.82 ± 0.17 | 8.30 ± 0.08 | 0.99 ± 6e−3 |
| | | WGAN | **1.13e−4** ± 1e−6 | 55.28 ± 0.72 | **75.19 ± 0.53** | **63.71 ± 0.57** | 40.16 ± 0.19 | 0.55 ± 4e−3 |
| | | DM | 9.23e−4 ± 3e−6 | **100 ± 0.00** | 1.20 ± 0.03 | 2.38 ± 0.06 | **40.35 ± 0.30** | **0.50** ± 1e−4 |
| | Multi CHRs (42409 SNPs) | VAE | 1.77e−2 ± 5e−5 | **100 ± 0.00** | 0.00 ± 0.00 | 0.00 ± 0.00 | 5.57 ± 0.07 | **0.50** ± 2e−3 |
| | | GAN | 1.46e−3 ± 5e−6 | **100 ± 0.01** | 0.65 ± 0.03 | 1.30 ± 0.05 | 5.10 ± 0.17 | 0.51 ± 7e−5 |
| | | WGAN | **1.63e−4** ± 1e−6 | 45.80 ± 0.42 | **64.35 ± 1.28** | **53.50 ± 0.46** | 19.58 ± 0.50 | 0.52 ± 1e−2 |
| | | DM | 9.56e−4 ± 3e−6 | **100 ± 0.00** | 1.00 ± 0.01 | 1.98 ± 0.02 | **20.04 ± 0.23** | **0.50** ± 8e−5 |

**Table 2.** Comparison of phenotype-prediction performance using real and synthetic genotype data.

| Dataset | | | XGBoost | | MLP | |
|---|---|---|---|---|---|---|
| | | | MSE ↓ | r ↑ | MSE ↓ | r ↑ |
| Cow | CHR 14 | Real | 0.60 ± 0.0054 | 0.65 ± 0.0034 | 0.67± 0.0088 | 0.61 ± 0.0066 |
| | | WGAN | 0.62 ± 0.0050 | 0.63 ± 0.0012 | **0.72 ± 0.0323** | **0.59 ± 0.0041** |
| | | DM | **0.61 ± 0.0056** | **0.64 ± 0.0023** | 0.74 ± 0.0188 | 0.55 ± 0.0033 |
| | CHR 5 | Real | 0.95 ± 0.0004 | 0.23 ± 0.0031 | 1.12 ± 0.0096 | 0.16 ± 0.0082 |
| | | WGAN | **0.95 ± 0.0014** | **0.23 ± 0.0021** | **1.14 ± 0.0168** | **0.15 ± 0.0151** |
| | | DM | 0.96 ± 0.0019 | 0.21 ± 0.0045 | 1.14 ± 0.0320 | 0.11± 0.0305 |
| | All CHRs | Real | 0.46 ± 0.0053 | 0.76 ± 0.0023 | 0.40 ± 0.0135 | 0.81 ± 0.0042 |
| | | WGAN | **0.52 ± 0.0069** | **0.72 ± 0.0027** | 0.49 ± 0.0261 | 0.75 ± 0.0067 |
| | | DM | 0.53 ± 0.0145 | 0.71 ± 0.0084 | **0.47 ± 0.0248** | **0.76 ± 0.0025** |
| Human | Ensembl | Real | 40.87± 0.1936 | 0.72 ± 0.0016 | 68.33 ± 6.1033 | 0.64 ± 0.0473 |
| | | WGAN | 43.44 ± 0.1392 | **0.70 ± 0.0011** | **69.87 ± 9.3012** | **0.59 ± 0.0105** |
| | | DM | **43.28 ± 0.6887** | **0.70 ± 0.0047** | 114.62 ± 31.7599 | 0.49± 0.0320 |
| | CHR 6 | Real | 42.81 ± 0.0791 | 0.71 ± 0.0006 | 88.53 ± 4.7244 | 0.44 ± 0.0384 |
| | | WGAN | **43.30 ± 0.1146** | **0.70 ± 0.0010** | **94.62 ± 5.2922** | **0.40 ± 0.0221** |
| | | DM | 44.65 ± 0.9555 | 0.69 ± 0.0079 | 218.95 ± 27.5067 | 0.24 ± 0.0099 |
| | CHR 12 | Real | 42.96 ± 0.0912 | 0.70 ± 0.0007 | 94.33 ± 5.6021 | 0.46 ± 0.0097 |
| | | WGAN | **43.41 ± 0.0684** | **0.70 ± 0.0006** | **96.52 ± 6.2831** | **0.42 ± 0.0158** |
| | | DM | 45.15 ± 1.4170 | 0.69 ± 0.0123 | 237.82 ± 14.8840 | 0.26 ± 0.0036 |
| | Multi CHRs | Real | 42.63 ± 0.1547 | 0.71 ± 0.0013 | 92.91 ± 3.2856 | 0.26 ± 0.0327 |
| | | WGAN | **43.44 ± 0.0800** | **0.70 ± 0.0006** | **96.51 ± 1.4907** | **0.23 ± 0.0334** |
| | | DM | 44.65 ± 0.9555 | 0.69 ± 0.0079 | 149.87 ± 5.7670 | 0.18 ± 0.0096 |

tion shows a higher correlation with the real population's β values compared to the DM-generated population.

Table 2 summarizes the predictive performance of machine learning and deep learning models on synthetic genotype to predict the conditioning phenotype, compared to the results from the real population. Similarly to the previous unconditional setting, for relatively small datasets (e.g., single chromosome in cow), both models achieve comparable performance to that obtained with real datasets. However, for more complex datasets, WGAN-generated synthetic genotype appears to better preserve the complex genotype-phenotype relationship, especially when using MLP as the prediction model. This aligns with WGAN's ability to improve the recall metric and more fully capture the distribution of real data. Overall, these results suggest that WGAN is able to generate synthetic population with genotype–phenotype association that closely mirror those observed in real data, as reflected by its consistently strong predictive performance across datasets and predictive model types.

## Discussion and Conclusion

The primary objective of this study was to investigate the effectiveness of widely used deep generative models for simulating genotype. We proposed specific adaptation for VAE, GAN, WGAN and diffusion models to better handle the discrete nature of genotype representation. Our experiments revealed that no single model performs best across all evaluation metrics and datasets. Each dataset exhibits distinct genetic properties, and we found that model performance is influenced by both the dimensionality of genotype sequence and the degree of SNP dependence. For relatively small and simple datasets (e.g., with a few thousand SNPs), we recommend using VAE due to its computational efficiency, training stability, and minimal hyperparameter tuning. For larger and more complex datasets with higher genetic diversity, WGAN-based model consistently outperforms the other models, particularly in capturing the overall distribution and the genotype-phenotype association.

We also proposed a comprehensive evaluation framework that combines multiple metrics to assess synthetic genotype quality from different angles. Since each metric captures a specific aspect, using them in combination provides a more complete evaluation. We found that not all previously developed metrics are robust. The *AA* score can produce misleading results in certain edge cases. Other metrics, such as the correlation score, are reliable but computationally intensive. Among all the metrics, recall stands out as a particularly valuable supervision signal during training, as it is more difficult to optimize and indicative of a model's ability to capture diversity. We recommend using PCA, $F_{ST}^{aggregated}$, precision, and recall during training to decide when to stop and only compute the more costly metrics afterward.

Our results are consistent with previous studies on haplotype generation, which have shown that generative models can accurately capture genetic structure. To our knowledge, this is the first work demonstrating that conditioning on phenotype enables generative models to generate synthetic populations that preserve genotype–phenotype associations. Such synthetic populations can be effectively applied to downstream tasks, such as GWAS, highlighting the potential of generative models to support genetics research. Furthermore, a trained generative model can function as a direct genotype–phenotype simulation tool, and the model itself can be

shared without releasing individual genomic sequences, thereby preserving privacy.

Several future research directions can be envisioned. In this study, we focused on models that learn the joint distribution of the entire genotype sequence, favoring a biologically grounded approach over sequential modeling. However, recent advances inspired by natural language processing, such as transformer-based models applied to DNA sequence [58–60], could also be adapted for genotype and merit further investigation. Another potential direction is the development of post-training refinement algorithms to improve the quality of generated sequences [61]. On the data side, future work could aim to better model additional features of genotype data, such as rare variants, population heterogeneity, and multi-phenotype conditioning. Incorporating modules that explicitly capture genotype–phenotype interaction could further enhance biological relevance. Lastly, exploring frugal learning strategies would be valuable, given the high dimensionality of genotype data and the computational demands of generative models.

## Availability of source code and requirements

The source code for the complete pipeline (model training, evaluation metrics, and experiments) is available on GitHub.

- **Project name:** DiscreteGenoGen
- **Project repository:** https://github.com/SihanXXX/DiscreteGenoGen
- **Operating system(s):** Platform independent
- **Programming language:** Python
- **Dependencies:** Listed in the `requirements.txt` file provided in the repository
- **License:** MIT License

## Data availability

Trained generative models for cows are available in our GitHub repository: https://github.com/SihanXXX/DiscreteGenoGen. These models can be directly used to simulate realistic genotype data and to reproduce our experiments. This supports the main practical motivation of our work: enabling genotype data sharing in a compact and privacy-preserving manner. A demonstration Jupyter Notebook for simulation is also provided at: https://github.com/SihanXXX/DiscreteGenoGen/tree/master/demo. Access to the UK Biobank dataset requires a separate application, which can be submitted at: https://www.ukbiobank.ac.uk/enable-your-research/apply-for-access.

## Abbreviations

VAE: Variational Autoencoder; DM: Diffusion Model; GAN: Generative Adversarial Network; SNP: Single Nucleotide Polymorphism; PCA: Principal Component Analysis; UMAP: Uniform Manifold Approximation and Projection; LD: Linkage Disequilibrium; ELBO: Evidence Lower Bound; GS: Gumbel–Softmax; WGAN: Wasserstein GAN; WGAN-GP: Wasserstein GAN with Gradient Penalty; GWAS: Genome-Wide Association Study; MLP: Multilayer Perceptron; AA: Nearest Neighbor Adversarial Accuracy; FST: Fixation Index; KNN: K-Nearest Neighbor; FC: Fat Content; YD: Yield Deviation; CHR: Chromosome;

## Additional Files

Supplementary material is available online.
**Supplementary Table 1.** Results of Principal Component Analysis (PCA) on genotype datasets.
**Supplementary Figure 1.** Illustration of a failed thresholding strategy when mapping generated continuous values to discrete genotypes.
**Supplementary Figure 2.** Exact genotype frequency matching using a thresholding strategy based on observed genotype frequencies.
**Supplementary Figure 3.** Precision and recall obtained with different distance metrics.
**Supplementary Figure 4.** Effect of SNP dependence on the difficulty of generative modeling.
**Supplementary Figure 5.** Detailed evaluation of the Nearest Neighbor Adversarial Accuracy (AA) score.
**Supplementary Figure 6.** Geometric illustration of a scenario where the AA score fails.

## Author Contributions

Data curation: SX, DB, TT. Conceptualization: SX, BH, JC, EB. Methodology: SX, BH, JC, EB. Formal analysis and investigation: SX. Writing—original draft: SX. Writing—review and editing: all authors. Supervision: BH, JC, EB.

## Funding

This work was supported by INRAE DigitBio Metaprogram.

## Acknowledgements

We thank Jocelyn De-Goër-De-Herve for managing the GPU infrastructure.

## Declarations

### Ethical Approval

Access to UK Biobank data was obtained under approved application 96326, and its use was conducted in accordance with all relevant guidelines and regulations. The acquisition of cow data was carried out in compliance with the ARRIVE guidelines.

### Consent for publication

Not applicable.

### Competing Interests

The authors declare that they have no competing interests.

## References

1. Reuter J, Spacek DV, Snyder M. High-Throughput Sequencing Technologies. Molecular Cell 2015;58(4):586–597.
2. Churko J, Mantalas G, Snyder M, et al. Overview of High Throughput Sequencing Technologies to Elucidate Molecular Pathways in Cardiovascular Diseases. Circulation research 2013 06;112:1613–23.
3. Gravel S. Population Genetics Models of Local Ancestry. Genetics 2012 06;191(2):607–619.
4. Kingman JFC. The coalescent. Stochastic Processes and their Applications 1982;13(3):235–248.
5. Hudson RR. Gene genealogies and the coalescent process. Oxford surveys in evolutionary biology 1990;7(1):44.
6. Kelleher J, Etheridge AM, McVean G. Efficient Coalescent Simu-

lation and Genealogical Analysis for Large Sample Sizes. PLOS Computational Biology 2016 05;12(5):1–22.
7. Hudson RR. Generating samples under a Wright–Fisher neutral model of genetic variation. Bioinformatics 2002 02;18(2):337–338.
8. Teshima KM, Innan H. mbs: modifying Hudson's ms software to generate samples of DNA sequences with a biallelic site under selection. BMC Bioinformatics 2009;10(1):166.
9. Baumdicker F, Bisschop G, Goldstein D, et al. Efficient ancestry and mutation simulation with msprime 1.0. Genetics 2021 12;220(3):iyab229.
10. Haller BC, Messer PW. SLiM 3: Forward Genetic Simulations Beyond the Wright–Fisher Model. Molecular Biology and Evolution 2019 01;36(3):632–637.
11. Ewing G, Hermisson J. MSMS: a coalescent simulation program including recombination, demographic structure and selection at a single locus. Bioinformatics 2010 06;26(16):2064–2065.
12. Peng B, Kimmel M. simuPOP: a forward-time population genetics simulation environment. Bioinformatics 2005 07;21(18):3686–3687.
13. Viñas R, Andrés-Terré H, Liò P, et al. Adversarial generation of gene expression data. Bioinformatics 2021 01;38(3):730–737.
14. Lacan A, Sebag M, Hanczar B. GAN-based data augmentation for transcriptomics: survey and comparative assessment. Bioinformatics 2023 06;39(Supplement_1):i111–i120.
15. Lacan A, André R, Sebag M, et al. In Silico Generation of Gene Expression profiles using Diffusion Models. bioRxiv 2024;.
16. Li Z, Ni Y, Beardall WAV, et al., DiscDiff: Latent Diffusion Model for DNA Sequence Generation; 2024.
17. Brixi G, Durrant MG, Ku J, et al. Genome modeling and design across all domains of life with Evo 2. bioRxiv 2025;.
18. Perera M, Montserrat DM, Barrabés M, et al. Generative Moment Matching Networks for Genotype Simulation. bioRxiv 2022;.
19. Geleta M, Montserrat DM, Giro-i Nieto X, et al. Deep Variational Autoencoders for Population Genetics. bioRxiv 2023;.
20. Montserrat DM, Bustamante C, Ioannidis A. Class-conditional vae-gan for local-ancestry simulation. arXiv preprint arXiv:191113220 2019;.
21. Nußberger J, Boesel F, Lenz S, et al. Synthetic observations from deep generative models and binary omics data with limited sample size. Briefings in Bioinformatics 2020 10;22(4):bbaa226.
22. Yelmen B, Decelle A, Ongaro L, et al. Creating artificial human genomes using generative neural networks. PLoS Genetics 2021 Feb;17(2):e1009303.
23. Yelmen B, Decelle A, Boulos LL, et al. Deep convolutional and conditional neural networks for large-scale genomic data generation. PLOS Computational Biology 2023;19(10):e1011584.
24. Szatkownik A, Furtlehner C, Charpiat G, et al. Latent generative modeling of long genetic sequences with GANs. bioRxiv 2024;p. 2024–08.
25. Szatkownik A, Planche L, Demeulle M, et al. Diffusion-based artificial genomes and their usefulness for local ancestry inference. bioRxiv 2024;p. 2024–10.
26. Kingma DP, Welling M. An Introduction to Variational Autoencoders. Foundations and Trends in Machine Learning 2019;12(4):307–392.
27. Goodfellow IJ, Pouget-Abadie J, Mirza M, et al. Generative Adversarial Nets. In: Advances in Neural Information Processing Systems, vol. 27; 2014. .
28. Ho J, Jain A, Abbeel P. Denoising Diffusion Probabilistic Models. In: Advances in Neural Information Processing Systems 33: Annual Conference on Neural Information Processing Systems 2020, NeurIPS 2020, December 6-12, 2020, virtual; 2020. .
29. Kynkäänniemi T, Karras T, Laine S, et al. Improved Precision and Recall Metric for Assessing Generative Models. In: Advances in Neural Information Processing Systems, vol. 32; 2019. .
30. Wold S, Esbensen K, Geladi P. Principal component analysis. Chemometrics and Intelligent Laboratory Systems 1987;2(1):37–52. Proceedings of the Multivariate Statistical Workshop for Geologists and Geochemists.
31. Jang E, Gu S, Poole B. Categorical Reparameterization with Gumbel-Softmax. In: 5th International Conference on Learning Representations, ICLR 2017, Toulon, France, April 24-26, 2017, Conference Track Proceedings; 2017. .
32. Kusner MJ, Hernández-Lobato JM. Gans for sequences of discrete elements with the gumbel-softmax distribution. arXiv preprint arXiv:161104051 2016;.
33. Bau D, Zhu J, Wulff J, et al. Seeing What a GAN Cannot Generate. In: 2019 IEEE/CVF International Conference on Computer Vision, ICCV 2019, Seoul, Korea (South), October 27 - November 2, 2019; 2019. p. 4501–4510.
34. Arjovsky M, Chintala S, Bottou L. Wasserstein Generative Adversarial Networks. In: Proceedings of the 34th International Conference on Machine Learning, vol. 70 of Proceedings of Machine Learning Research; 2017. p. 214–223.
35. Gulrajani I, Ahmed F, Arjovsky M, et al. Improved Training of Wasserstein GANs. In: Advances in Neural Information Processing Systems, vol. 30; 2017. .
36. Mirza M, Osindero S. Conditional Generative Adversarial Nets. CoRR 2014;.
37. McInnes L, Healy J, Saul N, et al. UMAP: Uniform Manifold Approximation and Projection. The Journal of Open Source Software 2018;3(29):861.
38. Laland KN, Uller T, Feldman MW, et al. The extended evolutionary synthesis: its structure, assumptions and predictions. Proceedings of the Royal Society B: Biological Sciences 2015;282(1813):20151019.
39. Wright S. THE GENETICAL STRUCTURE OF POPULATIONS. Annals of Eugenics 1949;15(1):323–354.
40. Weir BS, Cockerham CC. Estimating F-statistics for the analysis of population structure. Evolution 1984 Nov;38(6):1358–1370.
41. Slatkin M. Linkage Disequilibrium: Understanding the Evolutionary Past and Mapping the Medical Future. Nature Reviews Genetics 2008;9(6):477–485.
42. Rogers AR, Huff C. Linkage disequilibrium between loci with unknown phase. Genetics 2009 Jul;182(3):839–844.
43. Simonyan K, Zisserman A. Very Deep Convolutional Networks for Large-Scale Image Recognition. In: 3rd International Conference on Learning Representations, ICLR 2015, San Diego, CA, USA, May 7-9, 2015, Conference Track Proceedings; 2015. .
44. Uffelmann E, Huang QQ, Munung NS, et al. Genome-wide association studies. Nature Reviews Methods Primers 2021 aug;1(1):59.
45. Yale A, Dash S, Dutta R, et al. Generation and Evaluation of Privacy Preserving Synthetic Health Data. Neurocomputing 2020 Nov;416:244–255.
46. Sudlow C, Gallacher J, Allen N, et al. UK Biobank: an open access resource for identifying the causes of a wide range of complex diseases of middle and old age. PLoS Medicine 2015;12(3):e1001779.
47. Tribout T, Ducrocq V, Boichard D. HSSGBLUP: a Single-Step SNP BLUP genomic evaluation software adapted to large livestock populations. In: Proceedings of the 6th International Conference of Quantitative Genetics; 2020. p. 2–12.
48. Fernando RL, Cheng H, Golden BL, et al. Computational strategies for alternative single-step Bayesian regression models with large numbers of genotyped and non-genotyped animals. Genetics Selection Evolution 2016;48(1):96.
49. Littlejohn M, Tiplady K, Fink T, et al. Sequence-based Association Analysis Reveals an MGST1 eQTL with Pleiotropic Effects on Bovine Milk Composition. Scientific Reports 2016 04;6.
50. Winter A, Krämer W, Werner F, et al. Association of a lysine-232/alanine polymorphism in a bovine gene encoding acyl-

CoA:Diacylglycerol acyltransferase (DGAT1) with variation at a quantitative trait locus for milk fat content. Proceedings of the National Academy of Sciences of the United States of America 2002 08;99:9300–5.
51. Mullaney JM, Mills RE, Pittard WS, et al. Small insertions and deletions (INDELs) in human genomes. Human Molecular Genetics 2010 09;19(R2):R131–R136.
52. Lango Allen H, Estrada K, Lettre G, et al. Hundreds of variants clustered in genomic loci and biological pathways affect human height. Nature 2010;467(7317):832–838.
53. Anderson CA, Pettersson FH, Clarke GM, et al. Data quality control in genetic case-control association studies. Nature Protocols 2010;5(9):1564–1573.
54. Tahimic C, Wang Y, Bikle D. Anabolic effects of IGF-1 signaling on the skeleton. Frontiers in Endocrinology 2013 02;4:6.
55. He K, Zhang X, Ren S, et al. Deep Residual Learning for Image Recognition. In: Proceedings of 2016 IEEE Conference on Computer Vision and Pattern Recognition CVPR '16; 2016. p. 770–778.
56. Gao C, Huang K, Chen J, et al. Alleviating matthew effect of offline reinforcement learning in interactive recommendation. In: Proceedings of the 46th international ACM SIGIR conference on research and development in information retrieval; 2023. p. 238–248.
57. Ganev G, Oprisanu B, Cristofaro ED, Robin Hood and Matthew Effects: Differential Privacy Has Disparate Impact on Synthetic Data; 2022.
58. Ji Y, Zhou Z, Liu H, et al. DNABERT: pre-trained Bidirectional Encoder Representations from Transformers model for DNA-language in genome. Bioinformatics 2021 02;37(15):2112–2120.
59. Zhang D, Zhang W, Zhao Y, et al. DNAGPT: A Generalized Pre-trained Tool for Multiple DNA Sequence Analysis Tasks. bioRxiv 2024;.
60. Dalla-Torre H, Gonzalez L, Mendoza-Revilla J, et al. Nucleotide Transformer: building and evaluating robust foundation models for human genomics. Nature Methods 2025;22:287–297.
61. Li Z, Ni Y, Xia G, et al. Absorb & Escape: Overcoming Single Model Limitations in Generating Heterogeneous Genomic Sequences. In: Advances in Neural Information Processing Systems, vol. 37; 2024. p. 21949–21978.

Supplementary material file

Click here to access/download
**Supplementary Material**
supplementary.pdf

Cover letter
Click here to access/download;Personal Cover;cover_letter_giga_science.pdf

Dear Editor,

On behalf of my co-authors, I am pleased to submit our manuscript entitled *Learning Inherent Genetic Patterns and Trait Associations with Deep Generative Models for Discrete Genotype Simulation* for publication as a research article in GigaScience.

In this work, we investigate the use of widely adopted deep generative models to simulate realistic genotype data, both in unconditional and phenotype-conditioned settings. Compared to traditional statistical simulation tools, our approach offers several advantages. Specifically, our contributions include: 1. Architectural adaptations of neural networks for modeling discrete genotype sequences; 2. A comprehensive evaluation framework for assessing simulated genotype data from multiple perspectives; 3. Extensive experiments on large-scale cow and human datasets, ranging from single- to multi-chromosome settings. Our results demonstrate that deep generative models can effectively capture genetic variability and preserve genotype–phenotype associations. In addition, we provide a comparative analysis of model performance and the robustness of evaluation metrics, which we believe will be of interest to researchers in bioinformatics, machine learning, and genomics.

To promote reproducibility and open science, we have made the code publicly available. In addition, we have released the trained generative models, which enable others to directly simulate realistic genotype data. This supports the main motivation of our research: facilitating compact and privacy-preserving genomic data sharing, which aligns closely with GigaScience's mission to advance reproducible research, open data, and innovative computational approaches in the life sciences. For these reasons, we believe GigaScience is the most suitable journal for our work.

We have carefully reviewed the submission requirements on the journal's website and ensured that our research, writing, and formatting meet all of these guidelines. The manuscript compiles cleanly to the attached PDF file. We confirm that all authors have approved the final version of this manuscript for submission. The manuscript has not been published and is not under consideration elsewhere. A preprint has been posted on biorxiv, in line with common practice in this rapidly evolving field.

We thank you for considering our manuscript for publication in GigaScience and look forward to your response.

Sincerely,
Sihan Xie, on behalf of all co-authors

Graphical abstract file
Click here to access/download;Graphical Abstract;graphical abstract.pdf

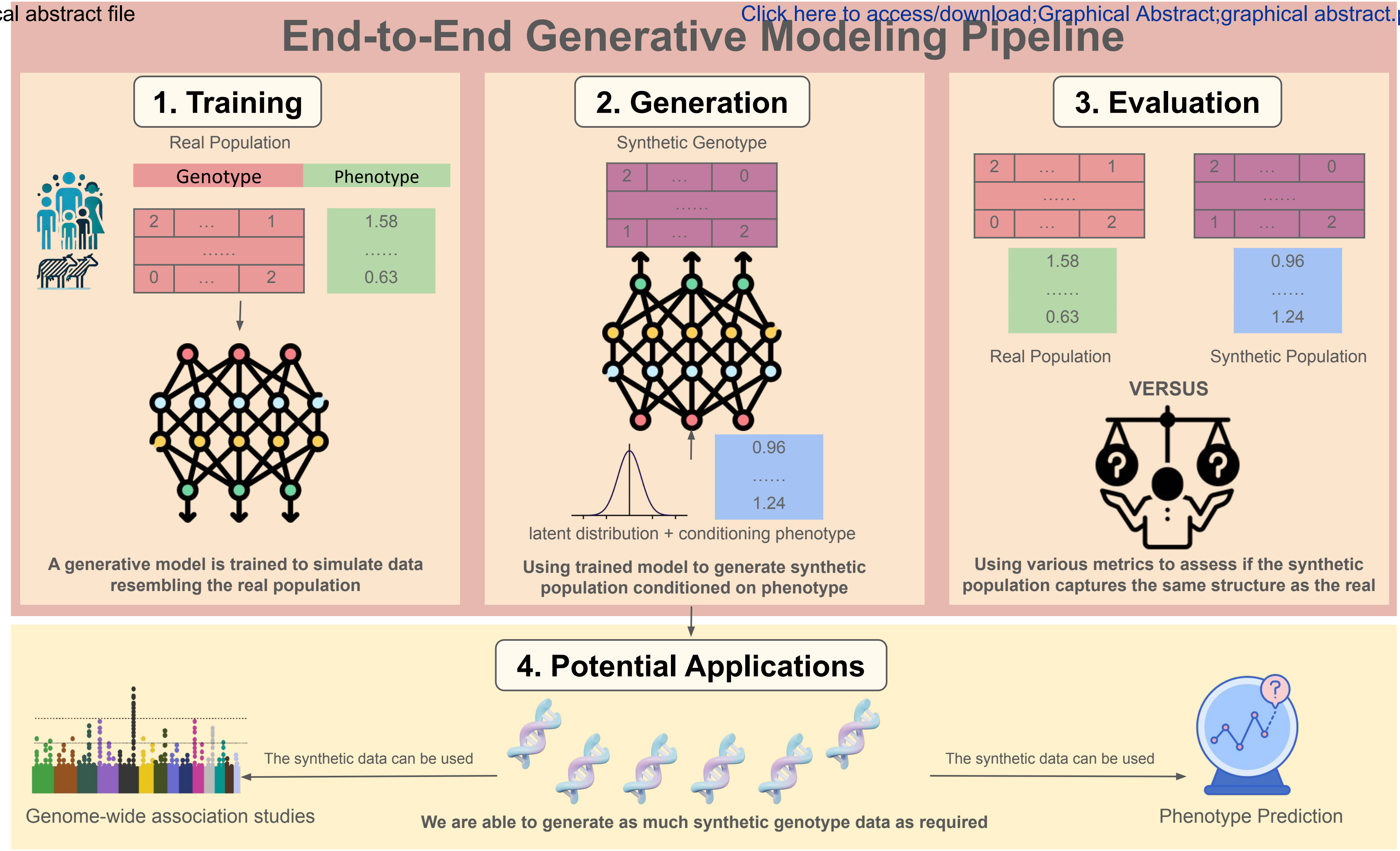

Supplement: giag044_GIGA-D-25-00343_Original_Submission [file giag044_giga-d-25-00343_original_submission.pdf]
