# Supplementary material for: Learning inherent genetic patterns and trait associations with deep generative models for discrete genotype simulation
Source: Gigascience. 2026 Apr 14;15:giag044. doi: 10.1093/gigascience/giag044 (PMC13163187; doi:10.1093/gigascience/giag044)
Supplement: giag044_Authors_Response_To_Reviewer_Comments_original_submission [file giag044_authors_response_to_reviewer_comments_original_submission.pdf]

Author's Response To Reviewer Comments

We thank the reviewer for the thorough and constructive assessment of our manuscript. We are very grateful for the positive comments regarding the clarity of the writing, the methodological comparison, the discussion, and the graphical abstract. We also apologize for not including line numbers during the review. We had assumed that the journal template should be used as provided for the submission. Below we provide a detailed, point-by-point response to all comments. All corresponding modifications have been incorporated in the revised manuscript. We have also included a PDF file containing our responses to the reviewers in the submitted files to facilitate your review.

To Reviewer #1:

1. I think it would be good to include a more thorough discussion of existing genotype-phenotype simulators in the non-generative setting (i.e. https://academic.oup.com/bioinformatics/article/35/19/3852/5341416). Possibly most people just use their own custom pipelines (i.e. simulate genotypes and then simulate the phenotype under the linear/logistic model), but it would be good to hear some discussion about the strengths of the author's approach relative to non-generative methods.

Thank you for this valuable suggestion. I am a regular user of SimuPOP (a Python library for population simulation), and from my personal experience I find that our method offers several advantages over traditional simulation approaches. In the original submission, our discussion of classical statistical simulators was indeed quite general. In the revised manuscript, we have expanded this section. We now highlight two main reasons why our approach differs from and improves upon traditional methods.
- Our method avoids a long multi-step pipeline and handles genotypes and phenotypes jointly. Many existing tools operate on haplotypes, whereas raw data obtained after genotype calling are genotypes. To perform genotype–phenotype simulations with traditional tools, one typically needs to phase genotypes into haplotypes (or generate synthetic haplotypes), simulate new haplotypes based on evolutionary process and mating scheme, reconstruct genotypes by collapsing haplotypes, and finally simulate phenotypes from the resulting genotypes. Our approach replaces this entire sequence with a single generative model that directly produces genotype–phenotype pairs.
- Our method does not rely on strong statistical assumptions and can learn non-linear genotype–phenotype relationship directly from the data. Traditional approaches often use linear models for continuous traits or logistic models for categorical traits, which require specifying SNP effect sizes and assume a fixed functional form. In contrast, our model learns the joint structure from the training data without imposing predefined relationships.

In Introduction section, we have added two new references (https://academic.oup.com/bioinformatics/article/35/19/3852/5341416 and https://academic.oup.com/bioinformatics/article/34/17/2951/4956348) for genotype-phenotype simulation and expanded the text to better compare our approach with traditional statistical simulation tools and previously developed haplotype-based generative models.

We have revised the first paragraph accordingly (page 1), now reading:
"Traditional statistical simulation methods are based on evolutionary models like Wright-Fisher model and coalescent theory, where users need to provide ancestral population and specify the entire evolutionary model. While these simulation tools are powerful, they often simplify various aspects of population genetics and evolutionary processes, which may not fully capture the complexities of real-world datasets."

The third paragraph (page 2) now reads:
“Directly simulating genotypes with generative models provides several advantages over traditional statistical methods and haplotype-based generative approaches. First, haplotype-based generative models offer limited conditioning capabilities, whereas our approach supports conditioning on phenotypes, enabling more flexible and application-oriented simulations. Second, traditional statistical workflows for simulating

genotype–phenotype pairs involve multiple steps and cannot generate both jointly in a single pass. A generative model consolidates these operations and produces genotypes and their corresponding phenotypes simultaneously. Traditional methods also rely on predefined statistical models to simulate phenotypes from genotypes, such as linear models for continuous traits or logistic models for categorical traits, which require specifying SNP effect sizes and impose a fixed functional form. In contrast, generative models do not make such assumptions and can learn non-linear genotype–phenotype relationship directly from the data. Finally, many haplotype-based simulators operate on small genomic regions with strong linkage disequilibrium, while our method supports genome-wide simulation. We demonstrate this capability in our cattle experiments, where we jointly model all 29 autosomes, thereby extending the scale of genomic simulation beyond the scope of previous methods.”

2. "Because genotype sequences lack inherent spatial or temporal structure, we avoided convolutional and recurrent layers." I don't really understand this, since SNPs are ordered along the chromosome. Are SNPs in LD filtered? It seems not since LD is one of the evaluation metrics///, and also "SNP dependency" is discussed but not clearly defined as something separate from LD or correlation patterns. Please be more clear about this - convolutional filters are frequently used in haplotype-based methods and I don't really see why they couldn't be used for genotypes here.

We thank the reviewer for raising this important point. Our original wording was indeed unclear. SNPs are ordered along the chromosome and exhibit LD structure, but their organization differs from the spatial or temporal dependencies typically modeled with convolutional or recurrent layers. Each SNP corresponds to a fixed physical position, and when SNPs are concatenated across chromosomes without encoding physical distances, the translation-invariance property of CNNs becomes difficult to justify. A pattern such as “010” may occur at several genomic positions, but these occurrences do not carry the same biological meaning, whereas convolutional filters would treat them as equivalent. Moreover, SNPs are irregularly spaced and they do not form a continuous sequence, which further limits the relevance of local convolutional operations.
Convolutional architectures have indeed been used in haplotype-based settings in the literature. However, in our previously published work on phenotype prediction using the same cow genotype dataset (Benchmarking predictive models: evaluating parametric, ensemble, and deep learning approaches for animal phenotype prediction from genotypes https://hal.em-lyon.com/SAPS/hal-04510253v1), we observed that MLPs outperformed CNNs. And more importantly, as discussed above, we consider fully connected layers biologically more appropriate for SNP data, since convolutional operations do not align well with how SNP markers are structured along the genome.

We have revised the manuscript to clarify this point and replaced the original sentence with a more precise explanation (page 5):
“All models were implemented using fully connected layers. Although SNPs are ordered along the chromosome, they do not form a continuous sequence and the same local allele pattern does not carry the same biological meaning at different positions. The translation-invariance assumption underlying convolutional layers is therefore not appropriate for SNP data. Likewise, SNPs do not exhibit temporal dependence, which makes recurrent architectures unsuitable. For these reasons, fully connected layers provide a more appropriate representation for genotype sequences.”

pg 1: "evolutionary parameters or provide ancestral population" in general the users need to specify the entire evolutionary model
Corrected to “where users need to provide ancestral population and specify the entire evolutionary model.”

pg 1: "population genetics" -> "evolution"?
In the original manuscript, we used the term population genetics in its broad sense, referring to the evolutionary processes acting on genetic variation (e.g., drift, selection, recombination, LD, and demography). However, to improve clarity, we revised the sentence to explicitly include both concepts. The updated version is: “While these tools are powerful,they often simplify various aspects of population genetics and evolutionary processes, which may limit their ability to capture the full complexity of real-

world datasets.”

pg 2: "can be shared publicly without directly exposing individual-level genetic information." Not all models would achieve this type of privacy guarantee.
We agree that generative models do not inherently guarantee privacy, as also illustrated in our experiments, and that privacy risks must therefore be explicitly evaluated. To avoid overstating the privacy protection offered by generative approaches, we have revised the sentence accordingly.
The updated version clarifies that only models that pass privacy-risk assessments should be considered suitable for sharing: “By shifting from explicit genomic sequences to generative models, the genome-wide training data itself remains private. The trained model can then be shared publicly if it passes an appropriate and comprehensive evaluation framework showing that it well reproduces population-level patterns without revealing individual-level genetic information.”

pg 2: precision and recall "have never been applied in previous haplotype generation studies" is this definitely true?
To the best of our knowledge, precision and recall, although commonly used in the generative modeling community, have not been applied in previous haplotype or genotype simulation studies, at least among the works we cite. However, in case we overlooked relevant work, we softened the statement in the manuscript.
It now reads:“precision and recall have been rarely used in previous haplotype generation studies.”

pg 3: "of synthetic population that mirror specific trait distribution." -> "of a synthetic population that mirrors a specific trait distribution."
Thank you for catching this.
We have corrected it as suggested.

Fig 3: legends unreadable
Figure 3 contains 16 subplots, which makes the text difficult to read at the scale shown in the manuscript. The original figure was exported at 600 dpi, so the text is readable when zoomed in. To improve readability in the revised version, we have adjusted the layout, increased the font size of the labels and legends, and clarified how to interpret the figure in the caption.

pg 6: "perfect correlation score" I wouldn't say this is perfect unless it is 1 (close but not quite)
Revised to “near-perfect”.

Fig 6: what are the units on the axes? (genetic distance?)
The axes do not represent genetic distance in Centimorgan, they correspond to SNP indices. Each unit on the x- or y-axis represents a single SNP, and the value at position (X, Y) is the pairwise LD between SNP X and SNP Y, computed using the scikit-allel Python library. This pairwise LD matrix is then visualized as a heatmap to highlight an LD block structure.

To make this clearer, we have revised the caption of Figure 6 to:
“Comparison of linkage disequilibrium between real and synthetic cow populations on chromosome 14 using LD block heatmaps. Each axis unit corresponds to a SNP index, and each cell represents the pairwise LD between two SNPs. The upper diagonal shows pairwise LD for the synthetic populations generated by (a) VAE, (b) GAN, (c) WGAN, and (d) DM, while the lower diagonal shows pairwise LD in the real population. This illustrates how well the model reproduces one representative LD block on cow chromosome 14.”

Note: We have carefully checked all uses of “genotype(s)” and “haplotype(s)” throughout the manuscript and corrected them as needed.

To Reviewer #2:

1. The diffusion model relies on PCA preprocessing, which may lose rare-variant information. Please quantify reconstruction error after PCA and discuss how this affects downstream GWAS signals.

The reconstruction error after PCA (retaining 90% of the variance) is reported in the Supplementary Materials (Table 1). The error is low (around 3%), which is why we use PCA to map discrete genotype sequences into a continuous latent space while keeping most of the relevant structure. This idea is similar to how VAEs are used in image-based latent diffusion models (Stable Diffusion), where a compact latent space is used before running the diffusion process. In our case, PCA offers a simple and efficient solution with low reconstruction error, and it avoids training an additional neural network.

To address the reviewer’s concern, we added a short discussion in the manuscript about the effect of PCA on rare variants. PCA captures the major axes of variation, but very low-frequency variants contribute little to the total variance and may be downweighted or lost in the reconstruction. This may slightly weaken their influence in downstream GWAS analyses. We now mention this limitation explicitly and comment on when PCA is appropriate (page 3). Now it reads:“Specifically, we projected genotypes into a lower-dimensional PCA space and trained the DMs in this continuous latent space. This single transformation yields three major benefits in one shot: it greatly reduces dimensionality and speeds up both training and inference; it transforms the discrete genotypes into a continuous representation that matches the assumptions of DMs; And it allows precise reconstruction via a simple linear multiplication. As with any latent-space compression method, some information loss is unavoidable. PCA preserves most of the global structure, but low-frequency variants that contribute little to the total variance may be reconstructed less accurately. This can slightly weaken downstream GWAS signals for rare variants, since the reconstructed genotypes may underestimate their true variation.”

2. The cow vs. human comparison is confounded by SNP density and LD structure. Please include LD-decay plots for both species and discuss how LD differences impact model performance.

We have added LD-decay plots for humans in the main article (Figure 4). We also expanded the discussion on how LD differences affect model performance. In cross-species comparisons, cattle populations typically have much stronger LD due to artificial selection and smaller effective population size, whereas humans show weaker LD. As a result, higher performance in cattle may simply reflect the fact that long LD blocks create more predictable patterns, while the shorter-range LD in humans makes the generative modeling task more challenging.

A similar effect appears within the human dataset: performance is better on chromosome 6 than on chromosome 12, even though chromosome 6 contains more SNPs. This can be explained by LD differences, since chromosome 6 has more high-LD SNP pairs, which provides clearer structure for the model to learn.

The revised text in the Results section now reads (page 6):
“Comparing across datasets, models consistently perform better on cow dataset than on human dataset. Cow population typically has much stronger LD due to artificial selection and smaller effective population size, whereas human population shows weaker LD (Figure 4). As a result, higher model performance in cow dataset likely reflects the fact that strong LD and high SNP dependency create more predictable patterns, while the greater genetic variability in human dataset increases the learning difficulty.”

3. The YD trait used for conditioning has undergone genetic pre-correction, which may partially embed the

signal the model is trying to learn. It would be helpful to repeat a single chromosome run using the raw, uncorrected milk-fat phenotype (or a simulated extreme percentile) and briefly comment on any qualitative differences in GWAS peaks or predictive r.

It is important to clarify that we did not apply any correction for genetic effects. The exact formula used for the pre-correction is provided in the Supplementary Materials Section 4. We view our pre-correction applied to the YD trait mainly as a way to remove environmental noise and batch-effect biases, rather than as something that hides the signal the model is expected to learn. In cattle, YD is measured across different herds, periods, and management conditions. These sources of variation can introduce substantial non-genetic or measurement bias. Correcting for such effects is standard practice in quantitative genetics, and traditional GWAS models would also include a fixed-effect term to account for them. For the human dataset, a similar correction would also be useful, although human height is less sensitive to measurement conditions than yield deviation. However, the metadata required for such correction of human dataset is not available for us.

4. Please provide a figure showing the Wasserstein-1 estimate (or discriminator loss) across training epochs for the best WGAN versus GAN run, along with a brief statement explaining how the smoother loss curve relates to the improved recall, thereby clarifying why WGAN performs better.

We have added the requested plots to Supplementary Materials (Figure 4). These figures show typical training dynamics for both WGAN and GAN, including the generator loss, the discriminator loss, and the evolution of precision and recall across epochs. The plots highlight the smoother and more stable behavior of the WGAN loss, which helps the model learn more consistently and leads to a steadier improvement in recall.
We also added a brief statement in the Results section of article (page 6):"However, GAN suffers from mode collapse and unstable training, leading to a recall score near 0. WGAN provides a clear improvement over GAN by stabilizing training, which allows the metrics to improve progressively."

5. The abbreviation "GS" for Gumbel-Softmax is listed in abbreviations but never used in the main text.
We have removed this unused abbreviation from the list.

6. Since the supplementary material is referenced in the main text as "Section 1, 2, 3," I recommend that supplementary figures also be explicitly cited as "Figure S1, S2," etc., in the main text.
Thank you for the suggestion.
We have updated the manuscript so that all supplementary figures are now explicitly referenced as "Figure S1", "Figure S2", etc. in the main text, consistent with the structure of the supplementary sections.

We thank the reviewers again for their thoughtful and constructive feedback. We believe the revisions have substantially improved the clarity and rigor of the manuscript. Should any additional comments arise, we would be happy to address them.

Cookie Preference Center

We use cookies which are necessary to make our site work. We may also use additional cookies to analyze, improve and personalize our content and your digital experience. For more information, see our Cookie Policy.

You may choose not to allow some types of cookies. However, blocking some types may impact your experience of our site and the services we are able to offer. See the different category headings below to find out more or change your settings.

Allow all

Manage Consent Preferences

Strictly Necessary Cookies

Always active

These cookies are necessary for the website to function and cannot be switched off in our systems. They are usually only set in response to actions made by you which amount to a request for services, such as setting your privacy preferences, logging in or filling in forms. You can set your browser to block or alert you about these cookies, but some parts of the site will not then work.

Functional Cookies

These cookies enable the website to provide enhanced functionality and personalisation. They may be set by us or by third party providers whose services we have added to our pages. If you do not allow these cookies then some or all of these services may not function properly.

Performance Cookies

These cookies allow us to count visits and traffic sources so we can measure and improve the performance of our site. They help us to know which pages are the most and least popular and see how visitors move around the site.

Targeting Cookies

These cookies may be set through our site by our advertising partners. They may be used by those companies to build a profile of your interests and show you relevant adverts on other sites. If you do not allow these cookies, you will experience less targeted advertising.

Cookie List

Clear

Apply Cancel
Consent Leg.Interest

Confirm my choices

Power
