# Supplementary material for: Learning inherent genetic patterns and trait associations with deep generative models for discrete genotype simulation
Source: Gigascience. 2026 Apr 14;15:giag044. doi: 10.1093/gigascience/giag044 (PMC13163187; doi:10.1093/gigascience/giag044)
Supplement: giag044_Reviewer_1_Report_original_submission — Reviewer 1 -- 12/16/2025 [file giag044_reviewer_1_report_original_submission.pdf]

Reviewer Report

Title: Learning Inherent Genetic Patterns and Trait Associations with Deep Generative Models for Discrete Genotype Simulation

Version: Original Submission **Date:** 12/16/2025

Reviewer name: Reviewer 1

Reviewer Comments to Author:

This paper adapts several popular generative methods for the problem of genotype-phenotype simulation. In particular they adapt VAE, GAN, WGAN, and diffusion models, overcoming the issue of discrete simulation. They have developed creative solutions for this problem, i.e. including a Gumbel-Softmax layer in the generator so GAN models can produce genotypes in {0,1,2} for each SNP.I found the manuscript overall well written and easy to follow, and the thorough comparison of the four methods was very well done. There was a good discussion at the end, including practical advice for which methods to use in different scenarios, as well as how to use the evaluation metrics during and after training. The limitations of the AA score (i.e. it can be 0.5 if the two components are 0 and 1, which is not the goal) were well presented, and I also liked the graphical abstract. I have a few more substantial comments and several minor fixes below.Note: please include line numbers if the manuscript is to be peer-reviewed!------------------Major comments------------------1. I think it would be good to include a more thorough discussion of existing genotype-phenotype simulators in the non-generative setting (i.e. https://academic.oup.com/bioinformatics/article/35/19/3852/5341416). Possibly most people just use their own custom pipelines (i.e. simulate genotypes and then simulate the phenotype under the linear/logistic model), but it would be good to hear some discussion about the strengths of the author's approach relative to non-generative methods.2. "Because genotype sequences lack inherent spatial or temporal structure, we avoided convolutional and recurrent layers." I don't really understand this, since SNPs are ordered along the chromosome. Are SNPs in LD filtered? It seems not since LD is one of the evaluation metrics, and also "SNP dependency" is discussed but not clearly defined as something separate from LD or correlation patterns. Please be more clear about this - convolutional filters are frequently used in haplotype-based methods and I don't really see why they couldn't be used for genotypes here.------------------Minor comments------------------Note: there are many cases when "haplotype" or "genotype" are used instead of "haplotypes" or "genotypes". For example: "We follow the PCA-based approach originally developed for haplotype" should be "haplotypes". Overall the paper could benefit from English-language editing, though the meanings of sentences are clear.pg 1: "evolutionary parameters or provide ancestral population" in general the users need to specify the entire evolutionary modelpg 1: "population genetics" -> "evolution"?pg 2: "can be shared publicly without directly exposing individual-level genetic information." Not all models would achieve this type of privacy guarantee.pg 2: precision and recall "have never been applied in previous haplotype generation studies" is this definitely true?pg 3: "of synthetic population that mirror specific trait distribution." -> "of a synthetic population that mirrors a specific trait distribution."Fig 3: legends unreadableNote: throughout it would be great to have a consistent color scheme for real/synthetic

data.pg 6: "perfect correlation score" I wouldn't say this is perfect unless it is 1 (close but not quite)Fig 6: what are the units on the axes? (genetic distance?)

Level of Interest

Please indicate how interesting you found the manuscript: Choose an item.

Quality of Written English

Please indicate the quality of language in the manuscript: Choose an item.

Declaration of Competing Interests

Please complete a declaration of competing interests, considering the following questions:

- Have you in the past five years received reimbursements, fees, funding, or salary from an organisation that may in any way gain or lose financially from the publication of this manuscript, either now or in the future?
- Do you hold any stocks or shares in an organisation that may in any way gain or lose financially from the publication of this manuscript, either now or in the future?
- Do you hold or are you currently applying for any patents relating to the content of the manuscript?
- Have you received reimbursements, fees, funding, or salary from an organization that holds or has applied for patents relating to the content of the manuscript?
- Do you have any other financial competing interests?
- Do you have any non-financial competing interests in relation to this paper?

If you can answer no to all of the above, write 'I declare that I have no competing interests' below. If your reply is yes to any, please give details below.

I declare that I have no competing interests

I agree to the open peer review policy of the journal. I understand that my name will be included on my report to the authors and, if the manuscript is accepted for publication, my named report including any attachments I upload will be posted on the website along with the authors' responses. I agree for my report to be made available under an Open Access Creative Commons CC-BY license (http://creativecommons.org/licenses/by/4.0/). I understand that any comments which I do not wish to be included in my named report can be included as confidential comments to the editors, which will not be published.

Choose an item.

To further support our reviewers, we have joined with Publons, where you can gain additional credit to further highlight your hard work (see: https://publons.com/journal/530/gigascience). On publication of this paper, your review will be automatically added to Publons, you can then choose whether or not to claim your Publons credit. I understand this statement.

Yes Choose an item.
